# Recombinant GM-CSF for diseases of GM-CSF insufficiency: Correcting dysfunctional mononuclear phagocyte disorders

**DOI:** 10.3389/fimmu.2022.1069444

**Published:** 2023-01-05

**Authors:** Hillard M. Lazarus, Katherine Pitts, Tisha Wang, Elinor Lee, Elizabeth Buchbinder, Michael Dougan, David G. Armstrong, Robert Paine, Carolyn E. Ragsdale, Timothy Boyd, Edwin P. Rock, Robert Peter Gale

**Affiliations:** ^1^ Department of Medicine, Division of Hematology and Oncology, Case Western Reserve University, Cleveland, OH, United States; ^2^ Medical Affairs, Partner Therapeutics, Inc., Lexington, MA, United States; ^3^ Division of Pulmonary, Critical Care, and Sleep Medicine, David Geffen School of Medicine at University of California, Los Angeles (UCLA), Los Angeles, CA, United States; ^4^ Department of Medicine, Harvard Medical School, Boston, MA, United States; ^5^ Department of Medical Oncology, Dana-Farber Cancer Institute, Boston, MA, United States; ^6^ Department of Medicine, Brigham and Women’s Hospital, Boston, MA, United States; ^7^ Division of Gastroenterology, Department of Medicine, Massachusetts General Hospital, Boston, MA, United States; ^8^ Keck School of Medicine, University of Southern California, Los Angeles, CA, United States; ^9^ Division of Respiratory, Critical Care, and Occupational Pulmonary Medicine, University of Utah, Salt Lake City, UT, United States; ^10^ Clinical Development, Partner Therapeutics, Inc., Lexington, MA, United States; ^11^ Hematology Centre, Department of Immunology and Inflammation, Imperial College, London, United Kingdom

**Keywords:** mononuclear phagocyte, sargramostim, immunomodulation, granulocyte-macrophage colony-stimulating factor, autoimmune pulmonary alveolar proteinosis (aPAP), COVID-19, wound healing, immune checkpoint inhibitors (ICI)

## Abstract

**Introduction:**

Endogenous granulocyte-macrophage colony-stimulating factor (GM-CSF), identified by its ability to support differentiation of hematopoietic cells into several types of myeloid cells, is now known to support maturation and maintain the metabolic capacity of mononuclear phagocytes including monocytes, macrophages, and dendritic cells. These cells sense and attack potential pathogens, present antigens to adaptive immune cells, and recruit other immune cells. Recombinant human (rhu) GM-CSF (e.g., sargramostim [glycosylated, yeast-derived rhu GM-CSF]) has immune modulating properties and can restore the normal function of mononuclear phagocytes rendered dysfunctional by deficient or insufficient endogenous GM-CSF.

**Methods:**

We reviewed the emerging biologic and cellular effects of GM-CSF. Experts in clinical disease areas caused by deficient or insufficient endogenous GM-CSF examined the role of GM-CSF in mononuclear phagocyte disorders including autoimmune pulmonary alveolar proteinosis (aPAP), diverse infections (including COVID-19), wound healing, and anti-cancer immune checkpoint inhibitor therapy.

**Results:**

We discuss emerging data for GM-CSF biology including the positive effects on mitochondrial function and cell metabolism, augmentation of phagocytosis and efferocytosis, and immune cell modulation. We further address how giving exogenous rhu GM-CSF may control or treat mononuclear phagocyte dysfunction disorders caused or exacerbated by GM-CSF deficiency or insufficiency. We discuss how rhu GM-CSF may augment the anti-cancer effects of immune checkpoint inhibitor immunotherapy as well as ameliorate immune-related adverse events.

**Discussion:**

We identify research gaps, opportunities, and the concept that rhu GM-CSF, by supporting and restoring the metabolic capacity and function of mononuclear phagocytes, can have significant therapeutic effects. rhu GM-CSF (e.g., sargramostim) might ameliorate multiple diseases of GM-CSF deficiency or insufficiency and address a high unmet medical need.

## 1 Introduction

Granulocyte-macrophage colony-stimulating factor (GM-CSF) was identified in the 1960s as a myeloid growth factor, purified in the 1970s, molecularly-cloned in the 1980s, and clinically developed in the 1990s (1). Sargramostim (Leukine^®^; Partner Therapeutics, Inc., Lexington, MA) is a glycosylated, yeast-derived recombinant human granulocyte-macrophage colony-stimulating factor (rhu GM-CSF), FDA-approved for 6 disease indications based on its safe and efficacious hematopoietic growth factor function, differing from human GM-CSF by one amino acid at position 23, where leucine is substituted for arginine (2). Its primary licensed use is for myeloid reconstitution after autologous or allogeneic blood and bone marrow transplantation (2). It is also used to shorten time to neutrophil recovery induced by chemotherapy for acute myeloid leukemia and as a medical countermeasure to treat people exposed to sufficient radiation to cause severe myelosuppression (2, 3). Here, we review emerging pleiotropic effects and therapeutic uses of GM-CSF and highlight results of recent and ongoing sargramostim clinical trials.

The hematopoietic growth factor medication class includes both rhu GM-CSF (*e.g.*, sargramostim) and rhu G-CSF; however, these products are not interchangeable. They differ in mechanism due to different receptors expressed on overlapping yet different target cells (4, 5). The G-CSF receptor is mainly expressed on neutrophils and bone marrow precursor cells, whereas the GM-CSF receptor is more broadly expressed on neutrophils, monocytes, eosinophils, and basophils.

Innovatively identifying and classifying diseases in terms of relative or absolute GM-CSF deficiency or insufficiency, and associated host cell dysfunction, have facilitated the recent investigations demonstrating the immunomodulatory functions of rhu GM-CSF on mononuclear phagocyte target cells (**Table 1**) (32). While beyond the scope of this paper, ongoing research in other disease states, such as neurodegenerative disorders, may also demonstrate potential effects of innate immune system modulation on patient outcomes (33–36). The mononuclear phagocyte system is a network of cells including monocytes, macrophages, and dendritic cells which are similar in their ability to sense and migrate to potential pathogens, cytotoxically engulf pathogens or dying cells, present antigens to adaptive immune cells, and secrete mediators to recruit additional immune cells (37). There is evidence that in diseases of GM-CSF deficiency and insufficiency, therapeutic use of exogenous rhu GM-CSF administration may augment mononuclear phagocyte function and correct for disease pathogenesis (28, 29, 38–67).

**Table 1:** Mechanisms and effects of GM-CSF deficiency or insufficiency disorders.

Disease	Mononuclear phagocyte*	Known or potential disorder mechanism	Known or potential effects of GM-CSF deficiency or insufficiency
aPAP	Macrophage	• GM-CSF deficiency results from neutralization by autoantibodies (6)	• Excess accumulation of surfactant proteins and lipids in alveoli (6) • Impaired differentiation of monocytes to macrophages that aid in protection and repair of damaged epithelial barriers (6, 7) • Decreased PPAR-γ expression leading to dysregulated cholesterol clearance in alveoli (6)
Infection	Monocyte, macrophage, dendritic cell	• GM-CSF deficiency or insufficiency may result from alveolar epithelial dysfunction as collateral effect of or direct infection with infectious pathogens impairing GM-CSF-secreting-type II alveolar epithelial cells (8, 9) Other stresses (*i.e*. oxidative stress) can lead to suppressed alveolar epithelial cell GM-CSF expression as well (10). • Pathogens overwhelm and dysregulate the immune system *via* either an overly pro-inflammatory response (hyperinflammation) or an overly anti-inflammatory response (immunoparalysis), leading to life-threatening organ damage (9)	• Decreased adaptive immune response *via* DC maturation and activation, and antigen-specific T cell recruitment (11) • Impaired alveolar macrophages (*e.g.*, in respiratory viral infections) lead to subsequent impaired opsonophagocytosis of pathogens and protection and repair of damaged epithelial barriers (12) • Impaired efferocytosis of necrotic inflammatory material (13)
Wound healing	Macrophage	• Relative GM-CSF deficiency results from many pathophysiological abnormalities inherent to underlying disease (*e.g.*, diabetes) (14, 15) • These abnormalities halt normal wound healing progression and spur ulcer development into chronic non-healing wounds (16, 17)	• Reduced neutrophil and macrophage chemotaxis and infiltration (15) • Decreased macrophage differentiation, efferocytotic function, PPAR-γ expression, and pro-inflammatory M1 to a pro-healing M2 phenotypic shift (18) • Delayed macrophage infiltration reduces lysed neutrophil clearance, causing tissue damage and prolonging the inflammatory healing phase, creating a chronically impaired healing milieu (17) • Insufficient macrophage actions impede granulation tissue formation, VEGF-dependent angiogenesis, and contractile myofibroblast differentiation delaying wound closure (19, 20)
Anti-cancer potential & irAE mitigation	Monocyte, dendritic cell	Anti-cancer potential: • ICI agent checkpoint receptor blockade (CLTA-4, LAG-3, PD-1, PD-L1) restores function to many antitumor immune cells that were actively suppressed by immune checkpoints, but does not restore function or enhance all antitumor immune cells necessary for optimal antitumor response (21). rhu GM-CSF (*e.g.*, sargramostim) may increase cytotoxic CD8^+^ T cell and dendritic cell recruitment to the tumor and sentinel lymph node, respectively (22–24). • Increased metabolic capacity of mononuclear phagocytes may counteract the immunosuppressive potential of tumor associated myeloid cells (25). irAE mitigation: • ICI-induced inflammation and immune response dysregulation may damage GM-CSF-producing lung tissue in checkpoint-induced pneumonitis and GI tract tissue in immune-mediated colitis (26, 27)	Anti-cancer potential: • Increased immune cell function (*e.g.*, ICI blockade of T cell anergy and exhaustion by tumor cells) without a concurrent boost in GM-CSF signaling, may create a relative GM-CSF deficiency and may result in suboptimal tumor-associated antigen presentation as studies have shown improved patient outcomes with ICI and sargramostim combination therapy (28, 29). irAE mitigation: • Based on similar immune cell populations and GM-CSF effects on epithelial barrier cells in inflammatory lung and GI tract disease (*e.g.*, influenza, aPAP, and Crohn’s), decreased endogenous GM-CSF may lead to unopposed inflammation due to reduce numbers of Tregs and MDSCs that dampen cytokine production, T cell proliferation, and chemotaxis that can damage the lungs and GI tract (6, 11, 25, 30, 31). • Decreased mucosal repair and recovery, decreased induction, and survival of DCs (11, 30)

aPAP: autoimmune pulmonary alveolar proteinosis; DC: dendritic cell; CTLA-4: cytotoxic T-lymphocyte-associated antigen 4; ICI: immune checkpoint inhibitor; irAE: immune-related adverse event; GI: gastrointestinal; LAG-3: lymphocyte-activation gene 3; MDSC: myeloid-derived suppressor cell; PD-1: programmed cell death protein 1; PD-L1: programmed cell death ligand 1; PPAR-γ: peroxisome proliferator-activated receptor gamma; Treg: regulatory T cell; VEGF: vascular endothelial growth factor.

*Mononuclear phagocytes include monocytes, macrophages, dendritic cells.

Three described rhu GM-CSF formulations differ in their glycosylation based on the expression system in which they’re produced (68, 69). Glycosylation in turn influences pharmacokinetics, biologic activity, and safety of each formulation. Molgramostim is produced in prokaryotic *Escherichia coli*, hence is not glycosylated, and regramostim is mammalian-derived from Chinese hamster ovary cells, hence has mammalian glycosylation; these two formulations are not commercially available (68, 69). Marketed sargramostim is yeast-derived with glycosylation similar to that of native GM-CSF (2). Of the three described rhu GM-CSFs, sargramostim glycosylation closely resembles that of native GM-CSF leading to comparable biologic activity, stability, resistance to degradation, tolerability, and immunogenicity (68, 69).

Sargramostim may be effective in multiple GM-CSF deficiency and insufficiency states. Sections to follow are organized by clinical disease area and information provided by experts in each therapeutic area who are studying sargramostim in clinical research. Sections include: emerging biology of GM-CSF, autoimmune pulmonary alveolar proteinosis (aPAP), infection, wound healing, and enhanced anti-cancer potential yet mitigation of immune checkpoint inhibitor immune-related adverse events.

## 2 Emerging biology of GM-CSF

In addition to myelopoietic actions, GM-CSF possesses anti-apoptotic effects and is reported to induce proliferation, mobilization, and activation of hematopoietic stem cells (70, 71), endothelial progenitor cells (6, 67), mesenchymal stromal cells (7, 8), pericytes (9), neural stem cells (72–76), and oligodendrocyte progenitor cells (76).

### 2.1 GM-CSF plays a crucial role in mitochondrial biogenesis and function

GM-CSF is crucial for mitochondrial maintenance in mononuclear phagocytes, as modeled in murine HIV studies (77). Furthermore, gene knockout animals reveal that GM-CSF influences mitochondrial turnover, function, and fatty acid β-oxidation (78). GM-CSF increases mitochondrial tricarboxylic acid cycle activity, oxidative phosphorylation, ATP production, and regulation of key metabolic pathways, such as glycolysis, pentose phosphate pathway, and amino acid synthesis. *Magmas* (mitochondria-associated granulocyte-macrophage CSF signaling molecule) is rapidly induced *in vitro* when murine myeloid-cell-line PGMD1 cells in culture are switched from IL-3 to GM-CSF in the medium (79). Also known as *Tim16* in mammals, *Pam16* in yeast, and *Blp* in drosophilia, *Magmas* is conserved across species, essential for cell growth, and anti-apoptotic when over-expressed (80–83). *Magmas* gene knockout mice die as embryos. RNAi-mediated knockdown of *Blp* resulted in mitochondrial membrane depolarization, 60% decreased ATP levels, 3.5-fold higher reactive oxygen species (ROS), cell-cycle arrest, autophagy activation, and 65% reduced cytochrome c oxidase activity in the mitochondrial electron transport chain (84). *Magmas* additionally functions as a ROS sensor and regulator, leading to reduced cellular ROS production (85).

### 2.2 GM-CSF supports efferocytosis

In addition to other effects that induce phagocyte populations (12, 86–90), GM-CSF supports efferocytosis, an energy intensive process in which macrophages engulf and digest apoptotic cells, such as short-lived tissue-infiltrating neutrophils, thereby preventing release or accumulation of necrotic inflammatory material (13). Decreased efferocytosis is associated with tissue necrosis and autoimmune disease (91). Opsonizing milk fat globule epidermal growth factor 8 (MFG–E8) bridges the “eat me” signal of phosphatidylserine displayed on apoptotic cell membranes with integrins αVβ5 and potentially αVβ3 on efferocytotic phagocytes (92). GM-CSF is required for expression of MFG-E8 by efferocytotic antigen-presenting cells (APCs) (93, 94) and induces integrins αVβ3 and αVβ5 (95, 96).

In addition, growth arrest-specific protein 6 (GAS6) bridges MerTK receptors on efferocytotic phagocytes to phosphatidylserine on the apoptotic cell external plasma membrane (91). Murine GM-CSF-induced bone marrow-derived macrophages express MerTK receptors and exhibit high phagocytic ability (97). Human macrophages differentiated with GM-CSF from healthy adult monocyte samples, and which were cultured either under growth/serum factor deficiency, or with subsequent treatment of IL-4 or incubation with apoptotic cells, result in M2-polarized macrophages that exhibit increased MerTK expression (98). Finally, activated peroxisome proliferator-activated receptor (PPAR)-γ and liver X receptor (LXR)-α drive anti-inflammatory macrophage engulfment of apoptotic cells (91). GM-CSF induces expression of LXRα in human blood mononuclear cells (99) and induces PPAR-γ expression in multiple myeloid cell types (100, 101).

### 2.3 GM-CSF modulates innate and adaptive immunity

GM-CSF broadly affects neutrophil biology *via* neutrophil induction (2), in particular by enhancing pro-survival effects (102). Oddly and reflective of cytokine pleiotropy, GM-CSF also facilitates auto-phagocyte-like neutrophil cell death (103). Also, GM-CSF downregulates chemotaxis *via* loss of signaling in response to Interleukin-8 (IL-8), the primary neutrophil chemotactic factor, whereas *N*-formyl-methyl-leucyl-phenylalanine (fMLP) chemotaxis is maintained (104). Finally, GM-CSF down-regulates neutrophil IL-8 receptor expression (105). In summary, GM-CSF might either facilitate or inhibit neutrophil chemotaxis depending on local environmental influences.

Separately, GM-CSF prevents blood neutrophil extravasation into tissues. L-Selectin mediates neutrophil trans-endothelial migration and is rapidly shed after activation and during the rolling phase of extravasation (106). ADAM17 (a disintegrin and metalloproteinase 17), also known as tumor necrosis factor-alpha-converting enzyme (TACE), is the principal “sheddase” that cleaves surface L-Selectin. Interestingly, ADAM17 sheddase activity acts on neutrophils but not monocytes. Consistent with its stimulation of ADAM17 expression, GM-CSF induces rapid, complete loss of L-Selectin, also known as leukocyte adhesion molecule-1 (LAM-1), from neutrophils, monocytes, and marrow cells but not lymphocytes (107).

Although its receptors are not expressed on lymphocytes, GM-CSF indirectly induces regulatory T cells (Tregs) in multiple autoimmune and chronic inflammatory disease models (108, 109). GM-CSF-deficient APCs exposed to MFG-E8-opsonized apoptotic cells produce altered cytokine profiles, resulting in decreased Tregs and increased inflammatory Th1 cells (93). GM-CSF also induces myeloid-derived suppressor cells (MDSCs) that suppress pro-inflammatory cytokine production, inhibit T cell proliferation, mediate chemotaxis, and activate Tregs (110–112).

In summary, GM-CSF influences a myriad of primarily myeloid cells, in part due to maturation and maintenance of metabolic capacity both systemically and locally, although the specific concentration that drives this change is yet to be determined (113). Follow-on effects include enhancement of phagocytosis and efferocytosis, as well as modulation of other immune cells including neutrophils and Tregs. Together, these data suggest that therapeutic rhu GM-CSF (*e.g.*, sargramostim) might generate benefit in diseases characterized by mononuclear phagocyte dysfunction or dysregulation.

## 3 Autoimmune pulmonary alveolar proteinosis (aPAP): A GM-CSF deficiency state

### 3.1 aPAP pathophysiology

High titers of neutralizing GM-CSF autoantibodies in aPAP lead to multiple effects that drive pathophysiology of this disease. These effects include reduced alveolar macrophage cholesterol clearance, impaired surfactant homeostasis, dysfunctional immune defense, and in a subset of patients, pulmonary fibrosis and end stage lung disease (6, 7, 114, 115). As reviewed above in **Emerging biology of GM-CSF**, GM-CSF deficiency impairs the expression of PPAR-γ, a key cholesterol regulator, leading to surfactant lipid accumulation within foamy alveolar macrophages (116, 117). GM-CSF autoantibodies also diminish neutrophil phagocytic antimicrobial functions and may lessen alveolar epithelial cell-derived GM-CSF activation and recruitment of alveolar macrophages, dendritic cells, and T cells (118). Reduced immune cell signaling and impaired gas exchange from surfactant accumulation contribute to increased incidence (13-25%) of opportunistic infections from organisms including *Aspergillus, Cryptococcus, Nocardia,* or *atypical mycobacteria* (7, 115, 119, 120). Because aPAP is a very rare disorder, the true prevalence of infection in this patient population and its associated mortality remain unclear.

Fibrosis, an uncommon but severe complication of aPAP, probably results from multiple mechanisms (114). Type II alveolar epithelial cells produce GM-CSF that aids in alveolar epithelial cell repair, leading to epithelial proliferation and barrier restoration (11, 118). In the presence of neutralizing GM-CSF autoantibodies, these homeostatic processes are impaired (6). Also, absence of GM-CSF results in lipid composition changes within the alveolar space that may lead to reduced synthesis of antifibrotic prostaglandin PGE2, which may enhance fibrogenesis, especially in the presence of additional insults (121, 122). The relationship between GM-CSF deficiency and fibrogenesis has been studied in murine models to date; human studies are needed yet are challenging in this rare disease (122). With the progression of pulmonary fibrosis, patients may develop severe, irreversible lung dysfunction for which the only known effective treatment is lung transplantation.

### 3.2 aPAP clinical investigations and gaps

Inhaled rhu GM-CSF is a potentially disease-modifying therapy with promising applications in aPAP, a mononuclear phagocyte disease. The inhaled route of administration delivers high drug concentrations directly to the disease site in the lung (123, 124). Clinical studies of inhaled rhu GM-CSF in aPAP have focused on achieving disease control or slowing or preventing disease progression. Trial endpoints have included measures of lung gas exchange, in particular diffusing capacity of the lungs for carbon monoxide (DLCO), exercise capacity, symptoms, and health-related quality of life. Inhaled rhu GM-CSF has been reported in clinical trials to improve clinical outcomes (42, 125). **Table 2** summarizes phase 2-3 studies of sargramostim and molgramostim that report benefit in achieving disease control for patients with aPAP. The adverse event data are reported in **Supplement Table S1**.

**Table 2:** Inhaled rhu GM-CSF phase 2-3 clinical studies in aPAP.

Study	Study design	rhu GM-CSF treatment	Results
Trapnell et al. (38) 2020	Prospective, randomized trial (N=138)	Molgramostim 300 μg inhaled daily, continuous or intermittently (every other week) x 24 weeks or placebo	Continuous molgramostim vs placebo: • Primary endpoint: Δ P(A-a)O_2_ from baseline: −12.8 mmHg vs −6.6 mmHg (*p*=0.03) o Δ in % predicted DLCO: 12.0 vs 4.2 o Δ SGRQ total score: -12.4 points vs -5.1 points
Tazawa et al. (39) 2019	Prospective, phase 2, randomized trial (N=64)	Sargramostim 125 μg inhaled twice daily x 7 days, every other week x 24 weeks or placebo	Sargramostim vs placebo: • Primary endpoint: Δ P(A-a)O_2_ from baseline: −4.50 mm Hg vs 0.17 mm Hg (*p*=0.02) • Δ in % predicted DLCO: 4.70 vs 0.37 • Δ CT density values: –22.4 HUs vs –2.5 HUs
Campo et al. (40, 41) 2016	Prospective, phase 2, randomized trial (N=18)	WLL followed by inhaled sargramostim 250 μg inhaled daily every other week x 12 weeks, then 250 μg daily x 2 consecutive days every 2 weeks x 6 months or WLL alone	Sargramostim + WLL, improvement at 30 months vs WLL alone • Significant improvement reported in sargramostim + WLL arm: (all (*p*<0.001): o Increased DLCO%: 15.7 o Increased FVC%: 11.8 o Increased TLC%: 10 o Increased FEV_1_%: 9.6 o Improved PaO_2_: 13.7 mmHg o Improved P(A-a)O_2_: -13.5 mmHg
Tazawa et al. (42) 2014	Prospective, phase 2, observational trial (N=35)	Long-term (30 month) follow up of Tazawa et al., 2010 study	Free from additional treatment vs additional treatment • Mean % predicted VC: 85.9 vs 71.6 (*p=*0.0045) • Mean % predicted FVC: 85.3 vs 71.4 (*p*=0.0064) • 23/35 patients did not require additional treatments • Median time to additional treatments (n=12): 50.5 weeks
Tazawa et al. (43) 2010	Prospective, phase 2, crossover, self-controlled, open-label trial (N=50)	• Observation period x 12 weeks • Sargramostim High dose period: 125 μg inhaled twice daily on days 1–8, no therapy on days 9–14 x six 2-week cycles (induction therapy) • Sargramostim Low dose period: 125 μg inhaled daily on days 1–4, no therapy on days 5–14 x six 2-week cycles (maintenance therapy)	Before vs after sargramostim therapy (observation vs high-dose induction + low-dose maintenance): • Primary endpoint: Δ P(A-a)O_2_ from baseline: -12.3 mmHg (*p*<0.0001) • Mean % predicted DLCO: 53.7 vs 61.4 (*p*=0.0008) • 6-min walk test: 393 meters vs 444 meters (*p*=0.0046)

DLCO, diffusing capacity of the lungs for carbon monoxide; FEV_1_, forced expiratory volume in 1 second; FVC, forced vital capacity; CT, computed tomography; HUs, Hounsfield units; PaO_2_, partial pressure of oxygen; P(A-a) O_2,_ alveolar arterial oxygen gradient; SGRQ, ST. George’s Respiratory Questionnaire; TLC, total lung capacity; VC, vital capacity; WLL, whole lung lavage.

Trapnell et al. and Tazawa et al. reported benefits utilizing inhaled molgramostim and sargramostim, respectively, compared to placebo in aPAP patients (38, 39). Campo et al. reported that sargramostim combined with whole lung lavage (WLL; the current standard of care for therapy in aPAP) was safe and more effective than WLL alone (40, 41). Also, in a case series of 5 patients with aPAP, Ohkouchi et al. (126) reported that inhaled sargramostim given after WLL significantly improved disease severity score parameters. These parameters included biomarkers such as mucin-like glycoprotein KL-6, carcinoembryonic antigen (CEA), and lactate dehydrogenase (LDH), as well as markers of oxygenation including alveolar-arterial oxygen gradient (A-aDO_2_) and partial pressure of oxygen (PaO_2_). Sargramostim given only before WLL did not improve these parameters. Although optimal dosing and duration of therapy have yet to be established, results of current ongoing research and real-world evidence are eagerly awaited (127). A disease-modifying therapy for aPAP is a high unmet need to slow disease progression, reduce infectious complications, and prevent pulmonary fibrosis and death.

### 3.3 Potential future developments in aPAP

Of the three rhu GM-CSF formulations described in **Introduction** (sargramostim, molgramostim, and regramostim), sargramostim is the only form that is currently commercially available. Sargramostim can be sourced from the United States and obtained globally through a “named patient program” per each nation’s healthcare governing body (128). Based on data mentioned here and additional case reports (126), sargramostim use may decrease healthcare utilization in this rare lung disease population. In a study evaluating 15 million people in the US from 2008 to 2012, patients with PAP were determined to have more outpatient visits (17.30 ± 13.77 vs 10.40 ± 11.38; *p* < 0.01), more emergency room visits (1.49 ± 1.17 vs 1.08 ± 0.27; *p* = 0.014), and longer hospital stays (15.96 ± 20.71 vs 5.40 ± 5.07 inpatient days; *p* = 0.027), compared with non-PAP controls (129). Annual per-patient healthcare costs were also 5-fold higher (approximately $40,000 more annually) for PAP patients than for non-PAP controls. Increased costs were attributed to disease-related treatments, including prescriptions, hospitalizations, and outpatient visits. In another retrospective cohort study of 500 U.S. patients admitted with a primary diagnosis of PAP between 2012 and 2014, mean actual cost per admission was $29,932 (CI: 13,739-46,124) with an overall annual cost burden of approximately $5 million (130).

Timely, accurate aPAP diagnosis also remains an issue due to disease rarity, low physician awareness, and limited access to the blood test for serum GM-CSF antibodies, which is performed in few centers worldwide. Testing centers include the CAP/CLIA certified lab at Cincinnati Children’s Hospital, National Jewish Health, the National Institutes of Health, and Cincinnati Children’s Hospital Pulmonary Alveolar Proteinosis Clinical Research Lab (131–133). Additional testing centers can be found in Japan, Germany, and China. Similar to other rare diseases, a patient advocacy organization has emerged (www.papfoundation.org) with the goal to unite the patient community and to connect patients with the specialist physician community for access to appropriate testing and relevant clinical trials.

Sargramostim is not approved by the FDA for use in aPAP, which limits access, reimbursement, and manufacturer ability to provide label information on safe and effective use in this setting. Also, broader sargramostim use in aPAP is limited by the absence of aPAP clinical consensus guidelines. Important questions to be addressed include impact and timing of treatment for asymptomatic or mildly symptomatic patients, as well as ideal dose and treatment duration for those with more severe disease. An ongoing international, multi-center, placebo-controlled trial of molgramostim will provide more information on dosing, efficacy, and safety data for the rhu GM-CSF agent class (127). Other aPAP treatment agents that upregulate PPAR-γ (*e.g.*, thiazolidinediones) or lower cholesterol (*e.g.*, statins) show preclinical promise and could deploy additional repurposed therapeutics available with known safety (134–136). Answers to these clinical questions and more are critical to patients and providers and will hopefully be elucidated *via* continued investigation of the potential of sargramostim and other therapies to modulate disease and prevent infection and fibrosis.

## 4 Immune responses to infections and risk of GM-CSF insufficiency

### 4.1 Infection pathophysiology

Mononuclear phagocyte dysfunction due to GM-CSF insufficiency can contribute to disease (*e.g.*, sepsis) precipitated by various events including trauma, major surgery, and hematopoietic cell transplant (HCT) (137, 138). Viral, bacterial, or fungal opportunistic infections all can cause sepsis and life-threatening organ dysfunction (139, 140). The focus of this section is viral respiratory pathogens and the delicate balance between an effective host response to eliminate respiratory viral infections *versus* an inadequate or even an overactive immune response to sepsis. The subset of patients who experience these inadequate or overactive immune responses may suffer serious or even fatal adverse events (8, 141, 142).

Insights garnered across dysfunctional mononuclear phagocyte disease states such as COVID-19, pneumonia, sepsis, and intensive care unit (ICU)-related critical illness may potentially be applied to many types of infections. Immunomodulatory agents (*e.g.*, sargramostim) that orchestrate the immune system and behaviors of immune cells for optimal host immune response to different pathogens may be beneficial in improving outcomes for many patients (58, 61, 62, 64, 65). While the hematopoietic growth factor rhu G-CSF (discussed in the **Introduction**) is more widely prescribed and comprises more than 95% of recombinant growth factor use, its use in infection has not demonstrated a mortality benefit in pneumonia when used in combination with antibiotic therapy (1, 4, 5, 143). G-CSF recruits and increases the number of neutrophils, whereas GM-CSF orchestrates the behavior of many innate and adaptive immune cells to combat pathogens while avoiding an overwhelming systemic response (4, 5). Viral infections and sepsis can be viewed as examples of mononuclear phagocyte dysfunction sequelae and will serve as models for further investigation of pathology, immune responses, and novel treatment strategies across patient populations to decrease morbidity and mortality.

#### 4.1.1 Respiratory viral infection

Respiratory pathogen transmission starts in the upper airway and occurs *via* direct physical contact, respiratory droplets, and/or airborne dissemination (141, 144). Specifically, viruses then incubate, replicate, and cause symptomatic infection. For immunocompetent patients, many acute respiratory infections are mild, self-limiting, and remain in the upper respiratory tract. For others, the infectious viral load can overwhelm and dysregulate the innate and adaptive immune systems, spread to the lower respiratory tract, and cause lung damage. This immune system dysregulation can ultimately cause life-threatening multiorgan dysfunction due to sequential failures in respiration, coagulation, liver function, cardiovascular status, and renal function (145).

In healthy lungs, alveolar macrophages, DCs, alveolar epithelial cells, and tissue-resident leukocytes continuously patrol and protect tissues from pathogens (118). Alveolar macrophages, which comprise more than 90% of lung leukocytes, are nurtured and controlled by alveolar epithelial cell signaling and phagocytose inhaled foreign particulates without triggering inflammation (8). During respiratory viral infection (likewise in bacterial and fungal infection), the microenvironment quickly changes to an inflammatory state (118). Alveolar macrophages and other lung-resident innate immune cells intercept the viral pathogen (8). Alveolar epithelial cells secrete chemokines and growth factors to recruit and activate neutrophils, monocytes, natural killer cells, and T cells for virus elimination. Lung-resident DCs are the main antigen-presenting cells (APCs) responsible for activating cytotoxic CD8^+^ and helper CD4^+^ T cells. Recruited short-lived neutrophils form and release neutrophil extracellular traps to capture viruses and halt viral spread. Neutrophils then undergo apoptosis and are removed by alveolar macrophages *via* efferocytosis, similar to neutrophil removal by tissue-resident macrophages as discussed in **Wound healing and risk of GM-CSF deficiency** (8, 17). Alveolar macrophages are often reduced in numbers in the lungs due to dysfunctional type II alveolar epithelial cells which are directly infected by both SARS-CoV-2 and influenza viruses (8, 9). Decreased alveolar macrophage function and numbers lead to dysregulated efferocytosis, prolonged inflammation, and tissue damage.

#### 4.1.2 Sepsis

**Figure 1:**
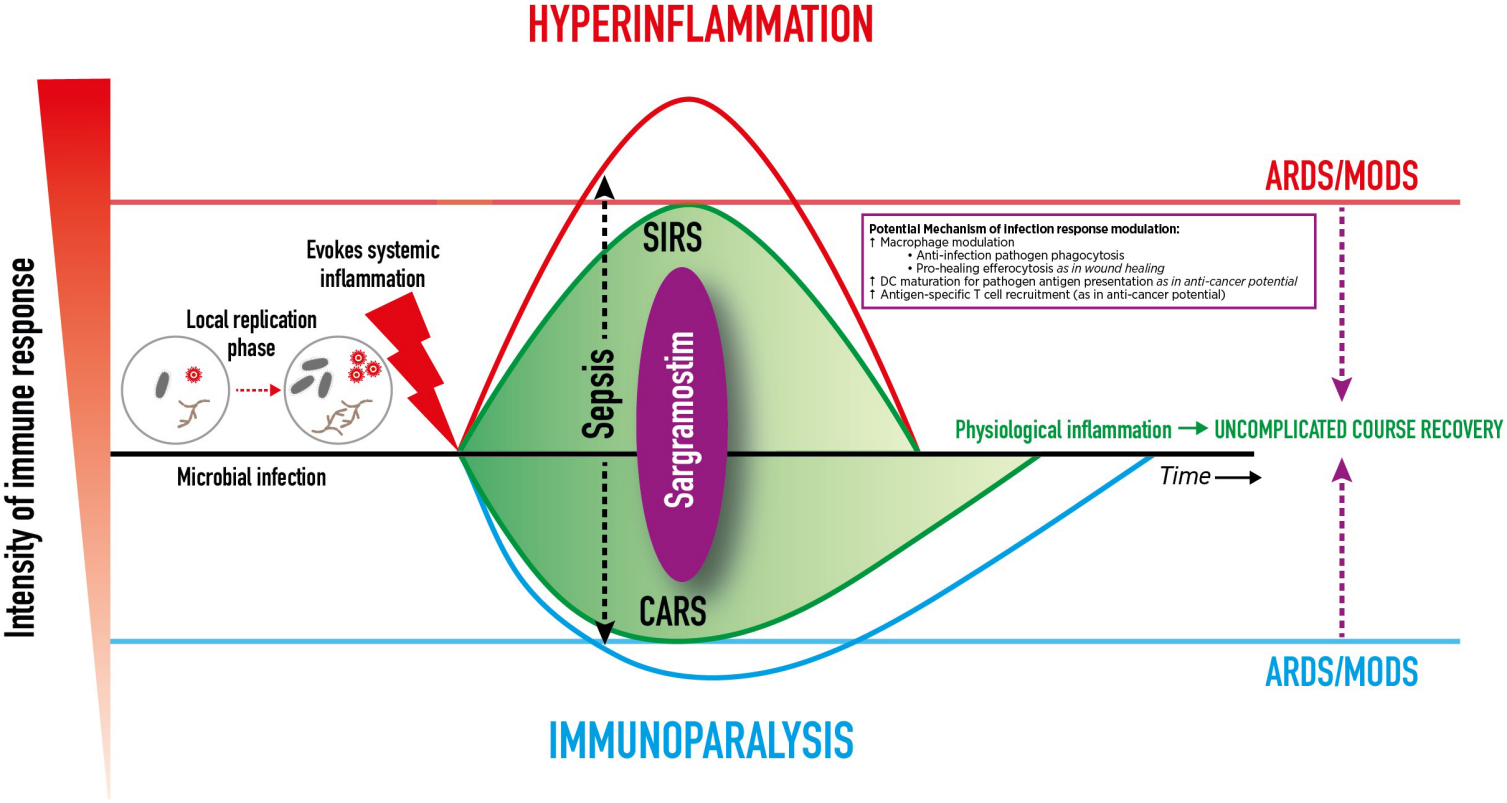
Dynamic Clinical Immune Response to Infection. Dynamic Clinical Immune Response to Infection and Potential Sargramostim Effect. After transmission, an infectious pathogen incubates, ***replicates***, and ***induces systemic inflammation***. A high pathogen load can overwhelm and dysregulate the innate and adaptive immune systems, spread, and cause life-threatening organ dysfunction. An overly pro-inflammatory immune response leads to ***systemic inflammatory response syndrome*** (SIRS).The ***compensatory anti-inflammatory response syndrome*** (CARS) slows the immune response. Simultaneous SIRS and CARS are considered normal complementary physiologic mechanisms that balance to restore homeostasis after infection onset. However, complications or dysregulated immune systems can incite excessive SIRS or CARS, skew the balance, and induce damage to vital organs (e.g., lungs, kidneys, heart, GI system, brain) and cause ***multiple organ dysfunction syndrome*** (MODS), and death. In the case of respiratory viral infections, damage to the lungs can result in ***acute respiratory distress syndrome*** (ARDS). ***Sargramostim*** (recombinant human granulocytemacrophage colony-stimulating factor) may mirror the effects of endogenous GM-CSF to modulate the immune response by alveolar macrophage activation, dendritic cell maturation, and antigen-specific T cell recruitment to aid in pathogen clearance. This may mitigate hyperinflammation and immunoparalysis to prevent ARDS and other organ damage.

An overly pro-inflammatory immune response leads to systemic inflammatory response syndrome (SIRS) with clinical features of fever, tachycardia, tachypnea, capillary leakage, and diffuse alveolar damage histology (146, 147). Subsequently, within minutes to hours of the pro-inflammatory response, the compensatory anti-inflammatory response syndrome (CARS) is initiated, which slows the immune response *via* downregulation of intracellular signaling (including internalization of HLA-DR on monocytes), transitioning the immune system to a hyporesponsive, immunosuppressive state (146, 147). In an immunocompetent patient, simultaneous SIRS and CARS are considered normal complementary physiologic mechanisms that balance one another to restore homeostasis after infection onset (**Figure 1**) (147). However, complications or dysregulated immune systems can incite excessive SIRS or CARS, and skew the delicate balance (147). The result may include inducing acute respiratory distress syndrome (ARDS) that potentially can progress to damage in other vital organs (*e.g.*, kidneys, heart, GI system, brain) leading to multiple organ dysfunction syndrome and death.

Infection-induced mononuclear phagocyte dysfunction, SIRS or CARS, may result in either hyperinflammation or immunoparalysis which in turn can progress to ARDS as influenced by disease severity, patient characteristics, pathogen or insult, and the physiologic inflammatory state (146). ARDS typically develops within 7 days of pneumonia or sepsis onset (142). In ARDS, edematous fluid accumulates within the interstitium and alveoli which may activate epithelial and endothelial cells, injure the microvasculature, impair gas exchange, and cause hypoxemia (8, 142). Alveolar macrophages recruit additional neutrophils which may lead to unmitigated-neutrophil release of inflammatory mediators, reactive oxygen species, and extracellular traps. Dysregulated inflammatory neutrophil activity may lead to a loss of pulmonary basement membrane integrity to further disrupt the epithelial-endothelial barrier and may promote ongoing dysfunctional endothelial or epithelial cell inflammatory mediator release, propagating the proinflammatory state (as seen in SARS-CoV-2 or influenza infection) (8, 142). Meanwhile, severe and prolonged CARS can result in a paralyzed immune system, sometimes termed “immunoparalysis.” Definitions for immunoparalysis vary and include: HLA-DR levels less than 8,000 monoclonal antibodies per cell in CD14^+^ monocytes; less than 30% of monocytes expressing HLA-DR; or a markedly decreased mononuclear phagocyte function that produce TNF-α in response to *ex vivo* challenge with lipopolysaccharide (65, 146) Also during CARS, B cells and DCs undergo apoptosis, T cells enter an exhausted state, and Treg and MDSC numbers increase (148).

Immunoparalysis and hyperinflammation due to infection can each result in ARDS, organ failure, and death, but their processes differ (149). In immunoparalysis, the immune response is suppressed such that pathogens are allowed to replicate and spread without challenge from the host immune system, leading to host cell damage, organ failure, and/or death (146, 150). Conversely, in hyperinflammation, overly activated immune cells, stimulated in response to causative pathogens, damage host cells *via* infiltration and exaggerates pathogen-mediated toxic substance release which can lead to organ failure and/or death (147).

An example of a unique population with iatrogenic mononuclear phagocyte dysfunction is immunocompromised HCT recipients receiving extremely immunosuppressive myeloablative preparatory agents (151, 152). Important endogenous pleiotropic cytokines, that are involved in the differentiation, maturation, and proliferation of host immune cells, are released as a compensatory mechanism after the ablation of the marrow (153, 154). After myeloablation, the immune system then responds *via* an outpouring of cytokines, like GM-CSF, in an attempt to stimulate bone marrow neutrophil production (155, 156). This immunosuppression and other predisposing factors heighten the risk of opportunistic infection, ARDS, and death in immunocompromised patients (157, 158). Other risk factors for immunocompromised HCT recipients include prior infections (viral, parasitic, fungal), immunosuppressive graft-*versus*-host disease prophylaxis agents that impair viral clearance (calcineurin inhibitors, corticosteroids), metabolic alterations, barrier defects, and qualitative and quantitative blood dyscrasias (neutropenia, lymphopenia, monocytopenia) (157). If infected, HCT recipients experience prolonged viral shedding and higher rates of upper respiratory infections often progressing to the lower respiratory tract (159, 160).

#### 4.1.3 GM-CSF in respiratory infection, ARDS, and sepsis-induced immunoparalysis

In healthy lungs, type II alveolar epithelial cells produce GM-CSF to aid in alveolar epithelial cell repair and restoration and to maintain surfactant homeostasis *via* alveolar macrophage cholesterol clearance (discussed in **Autoimmune Pulmonary Alveolar Proteinosis (aPAP): a GM-CSF deficiency state** and in the setting of checkpoint-induced pneumonitis in **Anti-cancer potential and mitigation of immune checkpoint inhibitor immune-related adverse events and risk of GM-CSF insufficiency**) (7, 11, 118, 125). GM-CSF is necessary for normal maturation and function of alveolar macrophages (6, 125). During lower respiratory viral infection, type II alveolar epithelial cells release GM-CSF to enhance the innate immune response of alveolar macrophage opsonophagocytosis of pathogens (as discussed in **Emerging biology of GM-CSF**) (11, 12). In studies in murine models, GM-CSF promotes adaptive immune responses *via* T cell, B cell, and DC maturation and activation that enable viral-specific antibodies production (161). After expansion and activation, GM-CSF facilitates lung-resident DCs’ migration to draining lymph nodes for additional antigen-specific T cell recruitment to improve viral clearance (162). Additional GM-CSF antiviral signaling may work in concert with interferon pathways (112).

Murine models have been very instructive for understanding the role of GM-CSF in respiratory viral infections. In transgenic mice lacking GM-CSF, survival after influenza infection was decreased due to impaired macrophage pathogen clearance (163). Conversely, transgenic mice with increased lung-GM-CSF expression experienced increased survival after influenza virus infection *via* enhanced alveolar macrophage activity (164). In another preclinical study, elevated alveolar GM-CSF concentrations in mice treated with intranasal recombinant murine GM-CSF increased alveolar macrophage numbers in bronchoalveolar lavage fluid (BALF) and improved survival after lethal influenza virus infection (165).

Release of antiviral pro-inflammatory immune response molecules into the systemic circulation can result in sepsis and can lead to ARDS (166). In animal models of post-viral ARDS, murine GM-CSF demonstrated immunomodulatory effects that improved the clinical response and symptoms associated with viral respiratory infections (167, 168). Increased airway GM-CSF expression and secretion in infected mice conferred a survival advantage in influenza-induced ARDS, attributed in part to the transition of pro-inflammatory M1 macrophages to the pro-healing M2 phenotype facilitated by GM-CSF (a similar transition discussed in **Wound healing and risk of GM-CSF deficiency**) (167). Inhaled recombinant murine GM-CSF improved locally-mediated murine-lung antibacterial resistance to systemic bacteremia during influenza infection (168). In adult patients with ARDS, elevated GM-CSF levels present in bronchoalveolar lavage fluid was associated with improved epithelial barrier integrity and survival (169).

In sepsis-induced immunoparalysis, impaired monocyte function leads to a diminished response to immune signaling, reduced pathogen phagocytosis, and reduced HLA-DR expression and thus a reduced ability to function as APCs (137, 170). As mentioned in **Wound healing and risk of GM-CSF deficiency,** HLA-DR is a class II MHC molecule typically found on APCs that links innate and adaptive immune responses *via* foreign antigen presentation to adaptive immune cells (*e.g.*, T cells) (25). In *in vitro* studies, GM-CSF has been shown to reverse sepsis-induced monocyte hyporesponsiveness by normalizing monocyte HLA-DR expression and subsequently improving pathogen antigen presentation to adaptive immune response cells to restore immunocompetence (171, 172). Timing and GM-CSF concentration may impact the degree of immune response (113). A study evaluated effects of rhu GM-CSF (molgramostim) and rhu G-CSF on HLA-DR expression in neonates with sepsis (n=60) *versus* healthy controls (n=41) (173). HLA-DR expression was decreased across all neonates with sepsis which was then progressively restored over 5 days. Normal values of HLA-DR expression were observed as early as day 1 for patients treated with molgramostim therapy, yet not until day 3 for patients treated with G-CSF and placebo. Molgramostim and sargramostim are both rhu GM-CSFs discussed in more detail in **Introduction** and **Autoimmune Pulmonary Alveolar Proteinosis (aPAP): a GM-CSF deficiency state**.

### 4.2 Infection immune response clinical investigation and gaps

Immunomodulatory agents that boost host immune function against different pathogens may be beneficial to many patients by improving oxygenation, preventing ARDS, and reversing immunoparalysis. When given *via* various routes (inhalation, intravenous, and subcutaneous), rhu GM-CSF has been reported to improve outcomes for patients who are critically ill, immunocompromised, and suffering from respiratory infection (**Table 3**) (58–65, 67). The adverse event data are reported in **Supplement Table S1**. Optimal rhu GM-CSF dosing, route of administration, and duration of therapy, however, have yet to be established. Several authors report sargramostim therapy benefited patients with these conditions. The studies, however, were small, used varying routes of rhu GM-CSF administration, and included infections from multiple or unknown pathogens; hence further investigations are needed.

**Table 3:** Use of rhu GM-CSF in respiratory viral infection, ARDS, SIRS, sepsis-induced immunoparalysis, and immune compromise.

Study	Study design & patient population	rhu GM-CSF treatment	Results	Impact on immune cells
Respiratory Viral Infection				
Paine et al. (174) 2022	Prospective, randomized, open-label trial (N=122) Non-ventilated hospitalized patients with COVID-19-associated hypoxemia	Sargramostim 125 μg inhaled twice daily x 5 days plus SOC vs SOC	Sargramostim + SOC vs SOC: • Improved oxygenation from baseline by day 6 (P(A-a)O_2_ gradient least squares mean change from baseline: -102.3 ± 19.4 vs -30.5 ± 26.9 mmHg; least squares mean difference: -71.7 ± 33.2 mmHg, *p*=0.033) • Lower proportion of patients requiring invasive mechanical ventilation by day 14 (11.5% vs 15.9%, *p*=0.49) • Improved 28-day all-cause mortality (11.5% vs 13.6%, *p*=0.76)	Sargramostim + SOC vs SOC: No significant increase from baseline in ferritin, D-dimer, and CRP, indicating sargramostim did not increase systemic inflammation
Bosteels et al. (58) 2022	Prospective, randomized, open-label trial (N=81) Non-ventilated hospitalized patients with COVID-19 and acute hypoxemic respiratory failure	Sargramostim 125 μg inhaled twice daily x 5 days plus SOC vs SOC	Sargramostim + SOC vs SOC: • Higher proportion of patients experienced at least 33% improvement in oxygenation (P(A-a)O_2_ gradient) from baseline by day 6 (54.3% vs 26.3%, *p*=0.0147)	Sargramostim + SOC vs SOC: Increase in circulating switched memory B-cells and CD38^+^ HLA-DR^+^ effector memory CD8^+^ T cells at day 5
ARDS				
Herold et al. (59) 2014	Single arm compassionate use study (N=6) Community-acquired pneumonia or ventilator-associated pneumonia with ARDS	Sargramostim 125 µg inhaled every 48 hours vs historical controls (n=4)	Sargramostim treatment vs historical controls: • Improved oxygenation (difference in slopes: 1.2 ± 0.4 [(PaO_2_/FIO_2_)/d, *p* = 0.0035] • Improved morbidity scores (improved SAPS scores from baseline, *p*=0.036)	Sargramostim vs historical controls: • Promoted alveolar macrophage M1 phenotype signifying successful delivery of drug to alveolar compartment • Increased alveolar mononuclear phagocyte activation measured by increased HLA-DR expression
Paine et al. (60) 2012	Prospective, phase 2 randomized, double-blind trial (N=130) Mechanically ventilated patients with ALI or ARDS. Primary sepsis (32.3% treatment group, 21.2% placebo group) and pneumonia (32.3% and 28.8%) were most common ALI/ARDS etiology	Sargramostim 250 µg/m^2^ IV infusion daily x 14 days vs placebo	Sargramostim vs placebo: • No difference in ventilator free days (10.8 ± 10.5 vs 10.7 ± 10.3 days, *p*=0.82) • No difference in 28-day mortality (17% vs 23%, *p*=0.31) • No difference in organ failure free days (15.7 ± 11.9 vs 12.8 ± 11.3 days, *p*=0.16)	Sargramostim vs placebo: • No significant increase from baseline in blood IL-6, IL-8 or TNF-α levels, indicating sargramostim did not increase systemic inflammation
SIRS				
Pinder et al. (61) 2018	Prospective, phase 2, randomized, single-blinded trial (N=38) ICU patients with SIRS and impaired neutrophil function (<50% phagocytic capacity)	Sargramostim 3 µg/kg SQ injection daily x 4 days vs placebo	Sargramostim vs placebo: • Higher proportion of patients with measured neutrophil phagocytosis ≥50% at day 2 (*p*=0.04) • Improved all-cause 30-day mortality (23.5% vs 28.6%, descriptive analysis only)	Sargramostim vs placebo: • Increased monocyte HLA-DR expression at day 2 (*p*<0.01)
Rosenbloom et al. (62) 2005	Prospective, randomized, unblinded trial (N=40) ICU patients with SIRS, and documented infection Solid-organ transplant recipients receiving standard immunosuppressive therapy (n=15) In sargramostim group (n=18): gram-positive infection (n=9), gram-negative infection (n=11), 1 with yeast infection (n=1), polymicrobial infection (n=4)	Sargramostim 125 µg/m^2^ continuous IV infusion over 72 hours (equivalent to 3 µg/kg/day) vs placebo	Sargramostim vs placebo: • Greater infection cure/improvement ratio (88% vs 36%; *p*=0.01) • No difference in rates of clinical resolution or mortality between solid organ transplant recipients and non-transplanted patients	Sargramostim vs placebo: • Increased monocyte HLA-DR expression to a level no different from healthy controls (*p*=0.27) • Positive correlation between HLA-DR expression and infection clearance (r=0.41; *p*=0.02) • Reversed the suppression and upregulated number of CD11b functional markers on circulating neutrophils and monocytes (*p*<0.01)
Presneill et al. (63) 2002	Prospective, phase 2, randomized, double-blind trial (N=18) Adults with sepsis-related SIRS and pulmonary dysfunction	Molgramostim 3 µg/kg IV infusion daily x 5 days + SOC or placebo + SOC	Baseline to day 5, molgramostim vs placebo: • Improved mean PaO_2_/FiO_2_ from baseline to day 5 in molgramostim group (136 ± 52 vs 185 ± 53, *p*=0.02) • Increased peripheral neutrophils (*p*=0.08)	Molgramostim vs placebo: • Increased neutrophil function and in treated group
Sepsis-induced Immunoparalysis				
Hall et al. (64) 2011	Prospective, randomized, open-label trial (N=14) Pediatric ICU patients with multiple organ dysfunction syndrome and immunoparalysis at high risk for nosocomial infection	Sargramostim 125 µg/m^2^ IV infusion daily x 7 days vs SOC	Sargramostim vs SOC: • No nosocomial infections observed (*p*<0.05) • No deaths observed • Fewer PICU days	Sargramostim treatment vs SOC: • Immunoparalysis reversed in < 7 days, *via* restored monocyte TNF-α response (*p*=0.001)
Meisel et al. (65) 2009	Prospective, randomized, double-blind trial (N=38) Patients with severe sepsis or septic shock with immunoparalysis [HLA-DR < 8,000 mAb/cell x 2 days]; Infections^*^: gram-positive (n=14), mixed gram-positive/gram-negative infection (n=12), gram-negative infection (n=8), fungal infection (n=3)	Sargramostim 4 µg/kg SQ injections daily x 5 days vs placebo On day 6: sargramostim increased to 8 µg/kg/day (if HLA-DR ≤15,000 mAb/cell at day 5) or maintained at 4 µg/kg/day (if HLA-DR > 15,000 mAb/cell)	Sargramostim vs placebo: • Shorter time of mechanical ventilation (148 ± 103 vs 207 ± 58 hours, *p*=0.037) • Improved APACHE II score from baseline (day 1, 21.3 ± 6.1 vs day 9, 16.7 ± 5.9, *p*=0.02 vs no difference day 1 to day 9 with placebo) • Shorter ICU stay (41 ± 26 vs 52 ± 39 days, *p*=NS) • Shorter intrahospital stay (59 ± 33 vs 69 ± 46 days, *p*=NS) • Similar 28-day mortality (16% vs 21%)	Sargramostim vs placebo: • Monocyte HLA-DR expression levels restored to normal range (19/19 patients vs 3/19 patients, *p*<0.001) • CD4^+^ and CD8^+^ T cells increased over time (*p*<0.05) and significantly higher at day 9 (*p*<0.05)
Bilgin et al. (66) 2001	Prospective, randomized trial (N=60) Neonates with sepsis-associated neonatal neutropenia	Molgramostim 5 µg/kg SQ injections daily x 7 days vs SOC	Molgramostim vs SOC: • Lower 28-day mortality (10% vs 30%, *p*<0.05)	Molgramostim vs SOC: • Increased day 7 absolute neutrophil count (8088 ± 2822/mm^3^ vs 2757 ± 823/mm^3^, *p*<0.01)
Immune compromise				
Wan et al. (67) 2015	Prospective, phase 4, randomized trial (N=206) Patients undergoing allogeneic HCT	Molgramostim 5-7 µg/kg SQ injection daily starting HCT day 5 until ANC ≥ 1.5 x 10^9^/L x 2 days vs G-CSF alone vs combination	Molgramostim-containing regimen vs G-CSF alone: • Lower 100-day transplant-related mortality (8.8% GM-CSF alone, 8.7% GM-CSF + G-CSF vs 21.7% G-CSF, *p*=0.034) • Lower 100-day cumulative mortality (10.3% GM-CSF alone vs 24.6% G-CSF, *p*=0.037) • Lower 600-day invasive fungal disease mortality (1.47% GM-CSF alone, GM-CSF+G-CSF 1.45% vs 11.59%, *p*=0.016) • Lower infection-related mortality rate (1.47% GM-CSF alone vs 14.49% G-CSF alone, *p*=0.011)	Molgramostim regimen vs G-CSF alone: • Higher circulating eosinophil levels between 3^rd^ to 5^th^ week after HCT (0.043 ± 0.093 vs 0.027 ± 0.021 x 10^9^/L, *p=*0.003) • Higher monocyte count in both molgramostim-containing regimens in 3^rd^ week after HCT (1.14 ± 0.317 vs 0.637 ± 0.580 x 10^9^/L, *p=*0.033)

ALI, acute lung injury; ANC, absolute neutrophil count; APACHE II, Acute Physiology and Chronic Health Evaluation; ARDS, acute respiratory distress syndrome; COVID-19, coronavirus disease 2019; CRP, C-reactive protein; FiO_2_, fraction of inspired oxygen; G-CSF, granulocyte colony-stimulating factor; GM-CSF, granulocyte-macrophage colony-stimulating factor; HCT, hematopoietic cell transplant; HLA-DR, human leukocyte antigen-DR isotype; IL-6, interleukin 6; IL-8, interleukin 8; ICU, intensive care unit; IV, intravenous; mAb, monoclonal antibody; NS, not significant; P(A-a)O_2_, alveolar-arterial gradient; PaO_2_, partial pressure of oxygen; PICU, pediatric intensive care unit; SAPS, simplified acute physiology score; SQ, subcutaneous; SIRS, systemic inflammatory response syndrome; SOC, standard of care; TNF-α, tumor necrosis factor alpha.

^*^1 patient in GM-CSF group died at study day 8 from sepsis-induced hemodynamic failure.

#### 4.2.1 Respiratory viral infection, ARDS, and SIRS

Rosenbloom et al. (62) reported greater infection cure/improvement ratio for sargramostim over placebo for infectious pneumonia, intra-abdominal, central nervous system, or blood stream infections of various microbial etiologies (*i.e*., gram-positive, gram-negative, yeast, and polymicrobial). In the setting of SARS-CoV-2 infection, Bosteels et al. (58) showed inhaled sargramostim improved oxygenation and alveolar gas exchange and increased numbers of circulating class-switched B cells and effector COVID-19-specific CD8^+^ lymphocytes. Studies have suggested efficacy and safety of inhaled sargramostim for COVID-19 treatment in hospitalized patients (174, 175).

Herold et al. (59) reported improved oxygenation with inhaled sargramostim for ARDS in hospitalized patients experiencing ARDS-pneumonia. Paine et al. (60) reported sargramostim treatment was found to be safe in patients with acute lung injury (ALI) and ARDS. The authors concluded sargramostim should continue to be studied in ARDS.

In a study conducted in critically ill patients with SIRS, Pinder et al. (61) reported decreased all-cause 30-day mortality with sargramostim compared to placebo.

#### 4.2.2 Sepsis-induced immunoparalysis and immunocompromised

Meisel et al. (65) reported decreased mechanical ventilation duration assessed at day 9, improved disease severity scores at day 9, and decreased length of ICU stay with subcutaneous sargramostim injections in patients manifesting immunoparalysis. CD4^+^ and CD8^+^ T cell numbers were increased, and HLA-DR levels were restored to normal levels as well. Additionally, Rosenbloom et al. (62) reported a positive correlation between HLA-DR expression and infection clearance after sargramostim therapy. Also, monocyte HLA-DR expression increased to levels no different from healthy controls. In pediatric ICU patients, Hall et al. (64) reported fewer pediatric ICU days, no deaths, and no nosocomial infections in patients who received sargramostim intravenous infusion.

In immunocompromised HCT recipients, Wan et al. (67) reported lower transplant-related mortality, lower cumulative mortality, lower invasive fungal disease mortality, and lower infection-related mortality in prophylactic molgramostim-containing regimens compared to granulocyte colony-stimulating factor (G-CSF). Additionally, therapeutic use of intranasal recombinant murine GM-CSF in immunosuppressed mice resulted in a decreased quantitative, PCR-assessed, fungal burden as compared to placebo (*p*=0.045) (176). In a new case series of invasive fungal disease in pediatric malignancy (n=15) and a systematic review of immunocompromised and immunocompetent patients (n=50), 92% and 82% of cases, respectively, showed a complete and/or partial response to invasive fungal disease when treated with adjunctive rhu GM-CSF in addition to standard of care (177).

Current and ongoing trials include important endpoints such as improvement in oxygen saturation, clinical indicators, PaO_2_/FiO_2_ ratio, and enhanced immunological effects, as well as improvements in major endpoints like reduction in mortality and days of hospitalization. Trials using an alternate route of rhu GM-CSF administration, specifically the inhaled route, may address availability concerns for respiratory treatments in both the inpatient and ambulatory settings (124). As learned from COVID-19 healthcare rationing, future investigations regarding rapid clinical responses using noninvasive direct pulmonary drug delivery with non-disease specific inhaled agents (*e.g*., sargramostim) may improve patient outcomes. Many disease-specific treatments such as monoclonal antibodies and antivirals, are virus- and/or variant-specific, limiting the potential patient population that could experience treatment benefit (178). The inhaled drug delivery technique, especially with versatile agents like sargramostim, may ultimately be demonstrated to be useful.

### 4.3 Potential future developments in infection immune response

There is enthusiasm for developing innovative therapies to improve patient outcomes after respiratory viral infections, ARDS, and sepsis-induced immunoparalysis. Beyond antimicrobial therapy, strategies include enhancing host defense by either replacing deficient cells (such as neutrophils in cases of cancer/chemotherapy-induced neutropenia) or potentially providing specially activated immune/inflammatory cells, akin to CAR-T cell therapy (179–181). Such treatments require enormous resources and in many instances (such as white blood cell transfusion for infection) have not shown clear benefit (182, 183). In contrast, sargramostim may safely target and modulate specific cells and cellular behavior to achieve an effective and efficient immune response (58, 59, 61, 62, 64, 65). Sargramostim’s actions on alveolar macrophages, dendritic cells, and T cells may allow for complementary immune response changes *versus* increases in cell numbers as seen with other agents such as rhu G-CSF (**Figure 1**) (5, 11, 12, 161, 162). Other novel products, such as hematopoietc stem cell-derived *ex vivo*-expanded myeloid progenitor cells and phenotypically typed functional granulocytes, have been shown to be efficacious in animal models (184, 185).

Biomarkers to predict and measure treatment effects in immunoparalysis, SIRS, CARS, and ARDS are urgently needed. Current surrogate biomarkers of inflammation (C-reactive protein [CRP], procalcitonin [PCT], ferritin) are used in practice, but further investigations of more sensitive and specific biomarkers are warranted (8). Definitions for immunoparalysis vary, for example monocyte HLA-DR levels less than 8,000 mAb/cell or TNF-α response assay to *ex vivo* stimulation results and should be standardized (64, 65). Standardized biomarkers would help stratify patients to better anticipate those at increased infection risk and hence should receive antimicrobial prophylaxis or treatment. Prospective immunophenotyping and/or patient stratification based on blood cell counts, immune function assays, cytokine levels, GM-CSF auto-antibody levels [*e.g.*, in *Cryptococcus gattii* cryptococcosis (186)], or other markers of inflammation would help define optimal timing of drug administration (*e.g.*, sargramostim) to prevent organ failure and death (9). Vulnerable patient populations such as immunocompromised HCT recipients, are at higher risk for all types of infection and should be a continued focus of future studies (138, 157).

Trials in pediatric sepsis [NCT03769844 (187), NCT05266001 (188)] aim to better understand the potential of sargramostim in modulating the immune system to enhance the pulmonary host defense capacity to eliminate pathogens, maintain alveolar homeostasis, and prevent disease progression. These trials will add to the existing evidence for sargramostim including attenuation of epithelial cell injury, epithelial repair, and improved barrier function and gas exchange in ARDS (59).

As the world continues to endure the COVID-19 pandemic, many trials investigating variant-independent treatment options are ongoing. Using single RNA-sequencing of MAFB and MAF transcription factors, 3 main lung macrophage populations expressing associated markers have been identified: FCN1 (ficolin-1; macrophages derived from circulating monocytes), SPP1 (secreted phosphoprotein 1; macrophage origin unknown), and FABP4 (fatty acid binding protein 4, an intracellular lipid chaperone and adipokine; found in GM-CSF-dependent alveolar macrophages) (118). These macrophage population ratios have shown to be altered in association with COVID-19 severity. Analysis of bronchoalveolar lavage fluid from patients infected with COVID-19 showed increased FCN1^high^ and SPP1^high^ macrophages and decreased FABP4^high^ macrophages correlated with disease progression (189). Altering the ratios of these lung macrophage populations towards an increased proportion of the FABP4^high^ macrophage subset, may promote pathogen clearance and epithelial repair while limiting an overly inflammatory response seen at the later stages of COVID-19 and other respiratory viral infections (189). Potential therapeutic intervention targeting the macrophage-activating upstream Jun N-terminal kinases (JNK) *via* MAPK inhibitors may alter macrophage population ratios, hence making these subsets both potential biomarkers and biopredictors (190).

The rapid SARS-CoV-2 genetic modifications created new variants that circumvented vaccine efforts and made it challenging to keep up with therapeutic targets due to changing resistance patterns. A trial in outpatients with COVID-19 is investigating using sargramostim to rebalance lung homeostasis to prevent disease progression to severe COVID-19 [NCT04707664 (191)]. Influenza shares many characteristics with SARS-CoV-2 as both pathogens invade and damage alveolar epithelial cells and have circumvented annual vaccine efforts, previously to an epidemic proportion. Although anticipated every year, seasonal influenza still causes significant morbidity and mortality, especially in high-risk HCT recipients (192). Prior to the COVID-19 pandemic, one study attributed influenza (seasonal A and B) to 30% of all respiratory viral infections in this population with up to 35% progressing to lower respiratory tract infections (193). In addition to HCT recipients, patients with other high risk factors (the very young, nursing home residence, chronic lung or heart disease, history of smoking) (194) contribute to the unmet need for further investigations to build upon the preclinical and clinical insights from sargramostim studies in COVID-19, pneumonia-associated lung injury, ARDS, and sepsis. Targeting these patient populations in future studies based on disease etiology, disease severity, and ideally key disease pathways per the individual patient will hone effective and efficient disease treatments and minimize side effects.

## 5 Wound healing and risk of GM-CSF insufficiency

### 5.1 Wound healing pathophysiology

**Figure 2:**
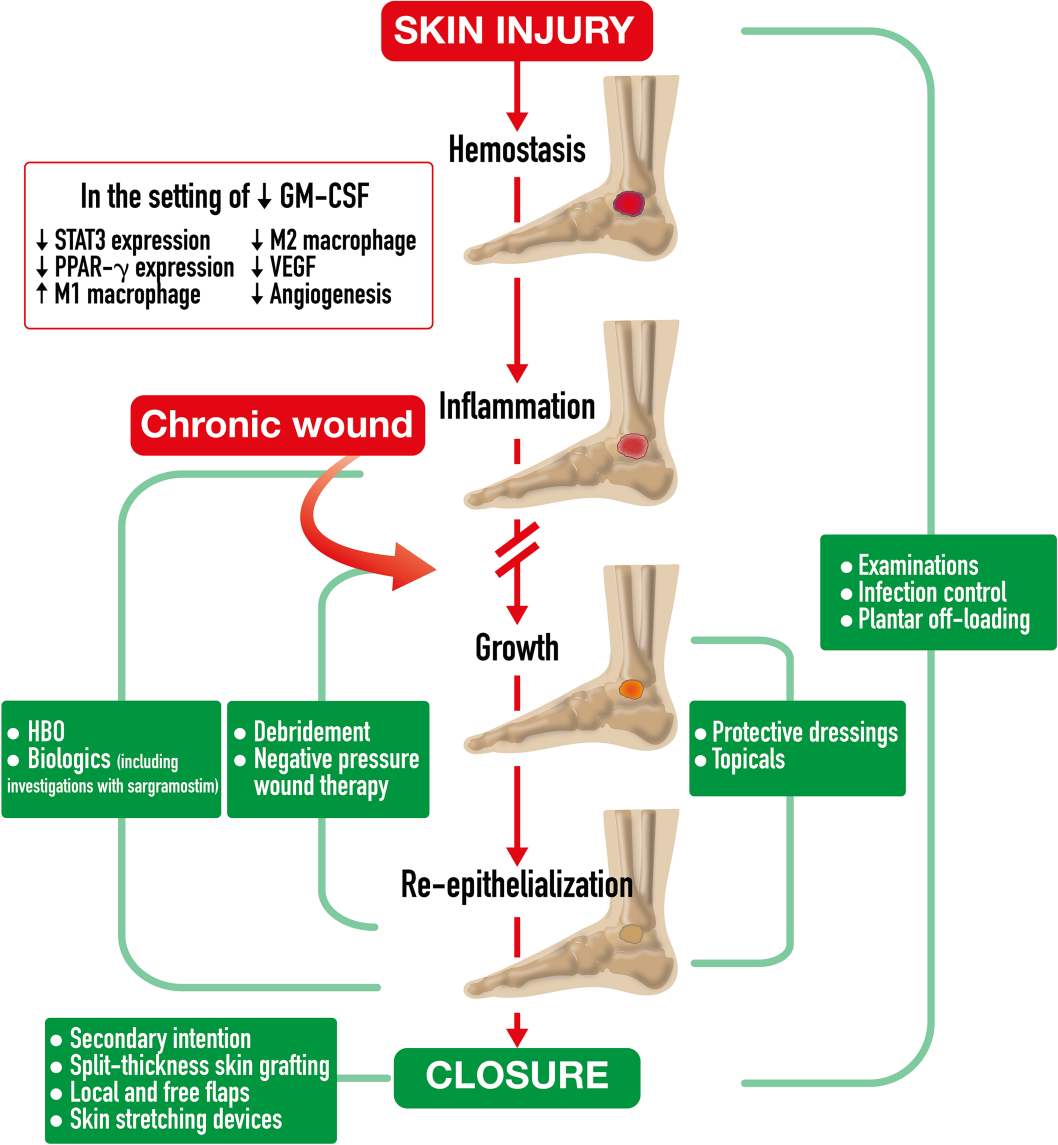
Chronic Wound Healing Process and Treatment Strategies. Chronic Wound Healing Process and Treatment Strategies. After spontaneous skin injury, the ***chronic wound healing process*** is comprised of four phases including hemostasis, inflammation, growth, and re-epithelization. This process often stalls at the inflammation stage in which GMCSF deficiency leads to reduced neutrophil and macrophage chemotaxis and infiltration, reduced signal transducer and activator of transcription 3 (STAT3) expression, insufficient macrophage differentiation, decreased efferocytotic function, impaired PPAR-g expression, and diminished pro-inflammatory M1 to pro-healing M2 transition. Insufficient macrophage actions impede granulation tissue formation, vascular endothelial growth factor (VEGF)-dependent angiogenesis, and contractile myofibroblast differentiation. All these factors ultimately delay wound closure. A continuum of ***treatment strategies*** for chronic lower extremity wounds is required for wound healing. Diverse strategies overlap to address key healing mechanisms. Strategies include wound bed preparation by debridement and negative pressure therapy, experimental immunologic modifications, and granulation tissue promotion with biologics such as sargramostim [recombinant human granulocytemacrophage colony-stimulating factor]), hyperbaric oxygen therapy (HBO), topicals, and protective dressings, as well as wound closure, including secondary intention, split thickness skin grafting, local and free flaps, and skin stretching devices. Examinations, antibiotic therapy, and plantar off-loading may be required at any phase.

Spontaneous acute wound healing in a normal host involves complex immune system interactions over time to restore the skin barrier after injury (17). Wound healing is comprised of four sequential and overlapping phases including hemostasis, inflammation, growth, and re-epithelization (**Figure 2**). Due to stresses both internal (*e.g.*, aging, genetics, nutrition, chronic disease) and external (*e.g.*, bacteria, medications), normal wound healing may be delayed or arrested at any stage (195). With concomitant diseases, such as diabetes, pathophysiologically inherent disease factors might further impair immune response, worsen peripheral arterial disease, and generate repetitive trauma due to neuropathic desensitization, all of which halt normal wound healing and contribute to development of chronic, non-healing wounds (*i.e.*, diabetic foot ulcer) (16, 17). Diabetic foot ulcers typically stall at the inflammation phase, partly attributable to accumulation of advanced glycation end products (AGEs) (196). Presence of AGEs leads to increased oxidative stress and inflammation, stiffer skin, and reduced innate immune cell adhesion. Also, inhibition of immune-cell-signaling p38/MAPK pathway results in decreased damaged cell removal and reduced primary skin cell (keratinocyte) migration (197).

Within hours of a spontaneous skin injury in an intact host, blood vessels constrict, and platelets form a fibrinogenic plug to stop bleeding to start the hemostasis phase (17). Local neutrophils and macrophages extravasate to the injury to defend against invading bacteria with cell recruitment increasing over 2 to 3 days (198, 199). Injured cells and mast cells release cytokines and other bioactive molecules to attract leukocytes, including Langerhans cells, dendritic cells, T cells, neutrophils, and monocytes (17, 195). Keratinocytes release endogenous GM-CSF that promotes local myeloid proliferation and supports additional inflammatory signaling (200, 201). A day after injury, neutrophils constitute half of all cells in the wound (17). In human diabetic foot ulcers, impairment in GM-CSF activation of signal transducer and activator of transcription 3 (STAT3) expression results in decreased immune cell recruitment (202). In a murine diabetes model, proinflammatory cytokines, such as interleukin 6 (IL-6), monocyte chemoattractant protein-1 (MCP-1), and GM-CSF, were reduced in wounds, compared to wounds in non-diabetic mice (15). Reduced signaling led to reduced neutrophil and macrophage recruitment and delaying healing. Mouse diabetic wound healing was almost completely restored by 2 weeks with perilesional exogenous rhu GM-CSF injected intradermally. In non-diabetic mice, exogenous rhu GM-CSF did not enhance wound healing.

In the inflammatory phase (3-20 days duration), immune cell recruitment continues, leading to pathogen, debris, and necrotic tissue removal (14). With an impaired wound healing environment, *i.e.* pressure ulcers in the elderly, decreased GM-CSF signaling leads to lower expression of nucleotide-binding domain-like receptor protein 3 (NLRP3) and reduced neutrophil interleukin-1 beta (IL-1β), resulting in impaired innate immune responses (203–205). In a normal host, GM-CSF signaling facilitates recruited monocyte differentiation into various immune response cells, including macrophages (206). Macrophages are often considered the most important immune cells in wound healing (19). They recognize and engulf pathogens, as well as eliminate expended neutrophils within 3 to 4 days of skin injury by efferocytosis (17). Decreased macrophage infiltration from impaired GM-CSF signaling in diabetes reduces neutrophil clearance, causing additional tissue damage from lysed neutrophils that prolongs the inflammatory phase. As mentioned in **Emerging biology of GM-CSF**, GM-CSF also induces PPAR-γ expression which is key to transitioning macrophages from a pro-inflammatory M1 phenotype to a pro-healing M2 phenotype (18). Impaired macrophage PPAR-γ activity and elevated M1 macrophages in diabetic wounds generate negative downstream effects. Decreased growth factor release, including vascular endothelial growth factor (VEGF) and platelet-derived growth factor (PDGF), reduces granulation tissue formation and prolongs inflammation both ultimately resulting in delayed wound closure (18, 207).

Within days to weeks from injury in a normal host, the growth phase begins in which granulation tissue formation and neovascularization occur (17, 208). Pro-healing M2 macrophages deposit extracellular matrix components, induce myofibroblasts, and phagocytose excess cells/matrix (17, 18). VEGF released by GM-CSF-stimulated macrophages acts as a key growth factor in early angiogenesis to promote micro-vessel sprouting (17, 209). VEGF and PDGF promote proliferation of keratinocytes, fibroblasts, and epithelial cells to create granulation tissue (207). GM-CSF also promotes maturation and stabilization of newly developed micro-vessels (capillaries) to establish new tissue blood supply (15). In diabetic foot ulcers, the necessary M2 macrophage population is decreased due to a dysregulated M1 to M2 phenotype shift, thereby delaying granulation and blood vessel formation (18).

In weeks to months, normal host re-epithelization occurs, and the wound closes (208). Normal connective tissue replaces granulation tissue while epidermal stem cells give rise to epidermal layers, hair follicles, and glands (17). GM-CSF-dependent M2 macrophages induce fibroblast differentiation into contractile myofibroblasts for wound closure (17, 19, 20, 210). Conversely, for patients with diabetes, fewer M2 macrophages result in decreased fibroblast differentiation and slower wound closure.

### 5.2 Wound healing clinical investigations and gaps

Clinical studies to date of rhu GM-CSF (*e.g.*, sargramostim or molgramostim) used diverse dosages, dosage forms, and durations of study therapy in small trials of diverse wound etiologies. Clinical studies of rhu GM-CSF in wound healing are summarized in **Table 4**. The adverse event data are reported in **Supplement Table S1**. Routes of administration have included both perilesional and topical, wound size and duration have varied considerably, and the affected subjects have varying degrees of immunocompromise. Hence, larger, randomized controlled trials that provide guidance on dose, route, frequency, and duration of therapy for definitive wound closure, specific to diabetes and other current etiologies, are needed to further confirm benefit of rhu GM-CSF.

**Table 4:** Clinical studies of rhu GM-CSF in wound healing.

Study	Study design & patient population	Wound	rhu GM-CSF treatment	Results
Venous Leg Ulcers				
Da Costa et al. (45) 1997	Prospective, randomized trial (N=25) Outpatient vascular surgery clinic patients	Chronic venous lower extremity ulcer (6 week to 5-year duration, 1-30cm^2^ surface area)	rhu GM-CSF 400 μg one-time perilesional subcutaneous injection or placebo	rhu GM-CSF vs placebo: • Higher proportion of patients had complete ulcer healing at 8 weeks (50% vs 11%) • Greater decrease in ulcer size at day 8 (mean -7.1cm^2^ vs +11cm^2^, *p*<0.005)
Da Costa et al. (44) 1999	Prospective, randomized trial (N=61)	Chronic venous leg ulcers (≥3 months duration, <30 cm^2^ surface area)	Molgramostim 200 μg or 400 μg perilesional subcutaneous injections weekly x max 4 weeks or until wound closure + SOC or SOC	Molgramostim-containing regimen vs placebo: • Greater proportion had complete ulcer healing at week 13 (57% in 200 μg group and 61% in the 400 μg group vs 19% in placebo group, *p*=0.014) • No ulcers recurred at 6-month wound evaluation
Cianfarani et al. (46) 2006	Single arm, prospective trial (N=8)	Chronic venous leg ulcer (2-12 years duration)	Molgramostim 150 μg perilesional intradermal injection x 4 simultaneous injections every other week	Molgramostim therapy: • Increased blood vessel density in the ulcer bed at day 5 vs day 0 (97.76 vessels/mm^2^ vs 59.69 vessels/mm^2^, *p*=0.017) • Did not increase vessel size • Increased expression of VEGF in the ulcer bed
Bianchi et al. (47) 2002	Prospective trial (N=5)	Chronic lower leg ulcers	Molgramostim 5 μg/mL topical solution, 1-2 mL applied topically three times daily x 1 week, then daily x 4 months	Molgramostim treatment: • Complete response in neuropathic diabetic ulcer (n=1) after 1 month of treatment • Vascular ulcers reported as no response (n=1), partial response of up to >50% wound healing (n=2), and complete response (n=1)
Diabetic Foot Ulcers				
Brem et al. (48) 2018	Case report (N=1)	Infected left great plantar toe diabetic ulcer (~1 year duration)	Sargramostim 500 μg intra- and perilesional injections during debridement weekly + SOC	Sargramostim treatment + SOC: • Complete wound healing after 5 weeks
Karlafti et al. (49) 2018	Case report (N=1)	Infected middle plantar surface diabetic foot ulcer (18-month duration, 5 cm diameter)	rhu GM-CSF 400 μg patch applied topically and injected once every 15 days x 2 months	rhu GM-CSF treatment: • Decreased ulcer diameter from 5 cm to 1.5 cm after 7 months of treatment • Full wound closure after 1 year
Thermal Burns				
Chi et al. (50) 2015	Prospective, randomized trial (N=30) Pediatric patients (age 1-5 years old)	Severe burns	rhu GM-CSF 100 μg/10g impregnated topical gel or placebo daily	rhu GM-CSF treatment vs placebo: • Faster time to healing (median 15 vs 19 days, *p*<0.05)
Yan et al. (51) 2017	Prospective, randomized trial (N=190 wounds, 95 patients)	190 deep, 2^nd^ degree burns wounds (each patient had at least 2 adjacent residual wounds >20 cm apart, <4 cm^2^ difference in size, and <25 cm^2^ surface area)	rhu GM-CSF gelatin, 1-2 mm applied topically daily x 28 days or placebo	rhu GM-CSF treatment vs placebo: • Faster healing (29.5% vs 19.9% decrease in wound size by day 7, *p*<0.001) • Shorter mean wound healing time (19 vs 26 days, *p*<0.001) • Greater granulation tissue capillary growth (11.29 vs 7.32 capillaries observed by day 14, *p*<0.001)
Pressure injuries				
Robson et al. (52) 2000	Prospective, randomized study (N=61) Hospitalized patients	Grade III/IV pressure ulcers (8 weeks duration, 10-200 cm^3^)	Molgramostim 2 μg/cm^2^ applied as topical spray daily x 35 days or rhu bFGF 5 μg/cm^2^ applied as topical spray daily x 35 days or Molgramostim applied as topical spray x 10 days followed by rhu bFGF applied as topical spray x 25 days or placebo	Molagramostim- or rhu bFGF-containing regimens vs placebo: • More patients experienced ≥ 85% decrease in ulcer volume at day 35 (*p*=0.03)
Payne et al. (53) 2001	Prospective, observational study (N=54) Hospitalized patients	Grade III/IV pressure ulcers (8 weeks duration, 10-200 cm^3^)	Long term follow-up study from Robson et al., 2000 study	More patients who experienced ≥ 85% healing within 35 days vs < 85% healing maintained complete wound closure at 1 year (84.6% vs 61%, *p*<0.05)

GM-CSF, granulocyte-macrophage colony-stimulating factor; rhu bFGF, recombinant basic fibroblast growth factor; SOC, standard of care; VEGF, vascular endothelial growth factor.

Other conditions have also been the subject of wound healing investigations. Case reports describe improved chronic wound healing with rhu GM-CSF treatment of patients with leukocyte or vascular dysfunction disorders, including glycogen storage disease (211), chronic granulomatous disease (211), common variable immunodeficiency (212), Klippel-Trénaunay-Weber syndrome (213), and cutaneous polyarteritis nodosa (214). Important issues to be addressed include optimal dose, route, schedule, and therapy duration within each wound etiology.

### 5.3 Potential future developments in wound healing

Deficiency of autocrine and paracrine GM-CSF activity in every stage of chronic wound healing illustrates a vital role for this cytokine. In a young, healthy host, endogenous GM-CSF is necessary for immune cell recruitment and maturation, phenotype shifts, keratinocyte proliferation, and angiogenesis. Successful wound repair and regeneration may well rely on timely activity of GM-CSF within healing wounds, especially its impact on macrophages, fibroblasts, and endothelial cells in rebuilding skin architecture.

Additional biomarkers to inform wound healing are needed. Vatankhah and colleagues (215) reported correlation of blood neutrophil-to-lymphocyte ratio with likelihood of diabetic foot ulcer nonhealing. Sawaya and associates (202) demonstrated that the diabetic foot ulcer immune cell landscape featured diminished GM-CSF activity with high and low proportions of monocytes and macrophages, respectively, indicating successful monocyte recruitment but deficient activation. Similar to blood monocytes, dermal stem cells of patients with diabetes mellitus also express low human leukocyte antigen-DR (HLA-DR), the receptor responsible for antigen presentation to CD4+ helper T lymphocytes and initiation of adaptive immune responses (216). As sargramostim is known to restore HLA-DR expression in post-surgery patients and sepsis-associated immunosuppression (as mentioned in **Immune responses to infections and risk of GM-CSF insufficiency** (65, 217), this receptor might become a clinically useful wound healing biomarker.

It soon may be possible to improve wound prevention of diverse etiologies, importantly including diabetic foot ulcers. Noninvasive Terahertz screening of diabetic foot skin dehydration estimates the amount of water that evaporates through skin to the external environment (218). Alternatively, thermometry (219) and point of care ultrasound imaging (220) are proposed to quantify risk of wound development. Topical barrier gels often serve as prophylaxis for potential post-spinal-cord injury pressure ulcers (221, 222). As mentioned in **Emerging biology of GM-CSF,** GM-CSF also may have protective neural cell effects (76, 223), hence a sargramostim gel could be a potentially interesting prophylactic approach.

Patients with chronic non-healing wounds have high morbidity and mortality, require multimodal healing treatment, and present an unmet need for novel therapies to improve outcomes (224). In the future, approaching all chronic wounds as a disease state, instead of as an underlying component of disease or the aging process, would establish a fresh viewpoint for management and prevention. Additionally, it is important to include elderly patients in future clinical trials as they are often excluded, as evidenced in cancer clinical trials (225, 226). Sargramostim’s immunotherapeutic potential should be further studied in at-risk patients and address wound healing outcomes that optimize ulcer-free, hospital-free, and activity-rich days. Sargramostim’s preventative therapeutic potential should be further studied, especially in combination with improved noninterventional diagnostic practices.

## 6 Anti-cancer potential and mitigation of immune checkpoint inhibitor immune-related adverse events and risk of GM-CSF insufficiency

### 6.1 Pathophysiology of antitumor effects

In a healthy immune system, immune checkpoint receptors dampen immune responses to prevent prolonged T cell activation and autoimmunity (227). Immune checkpoint inhibitors (ICIs) block checkpoint-originated immune suppression both in the tumor, to enable a desired antitumor T cell response, and potentially in healthy organ systems such as the gastrointestinal (GI) tract and lungs, causing unwanted tissue damage. Immune-related toxicity can occur at any time during treatment, is a frequent cause of ICI discontinuation, and may persist after cessation of therapy (228).The GI and pulmonary organ systems provide dual immune protection *via* physical barriers and cell-mediated immune responses. Immune-mediated colitis and checkpoint-induced pneumonitis can resemble infectious or spontaneous autoimmune disorders, and may have a delayed clinical presentation creating a diagnostic challenge for clinicians (228).

Immunotherapy aims to mobilize the immune system and restore antitumor immunity that is actively suppressed either by tumors cells themselves or by other immune cells in the tumor microenvironment (21). In an intact immune system, immune checkpoint receptors like CTLA-4, LAG-3, and PD-1 inhibit T cell activity at different steps of the immune response (229, 230). These receptors assist in limiting and preventing autoimmunity that could occur with unencumbered activated T cells. Blocking these receptors with ICIs inhibits inactivation, allowing the activated immune system to overcome cancer escape mechanisms and eliminate tumor cells (230–232).

**Figure 3:**
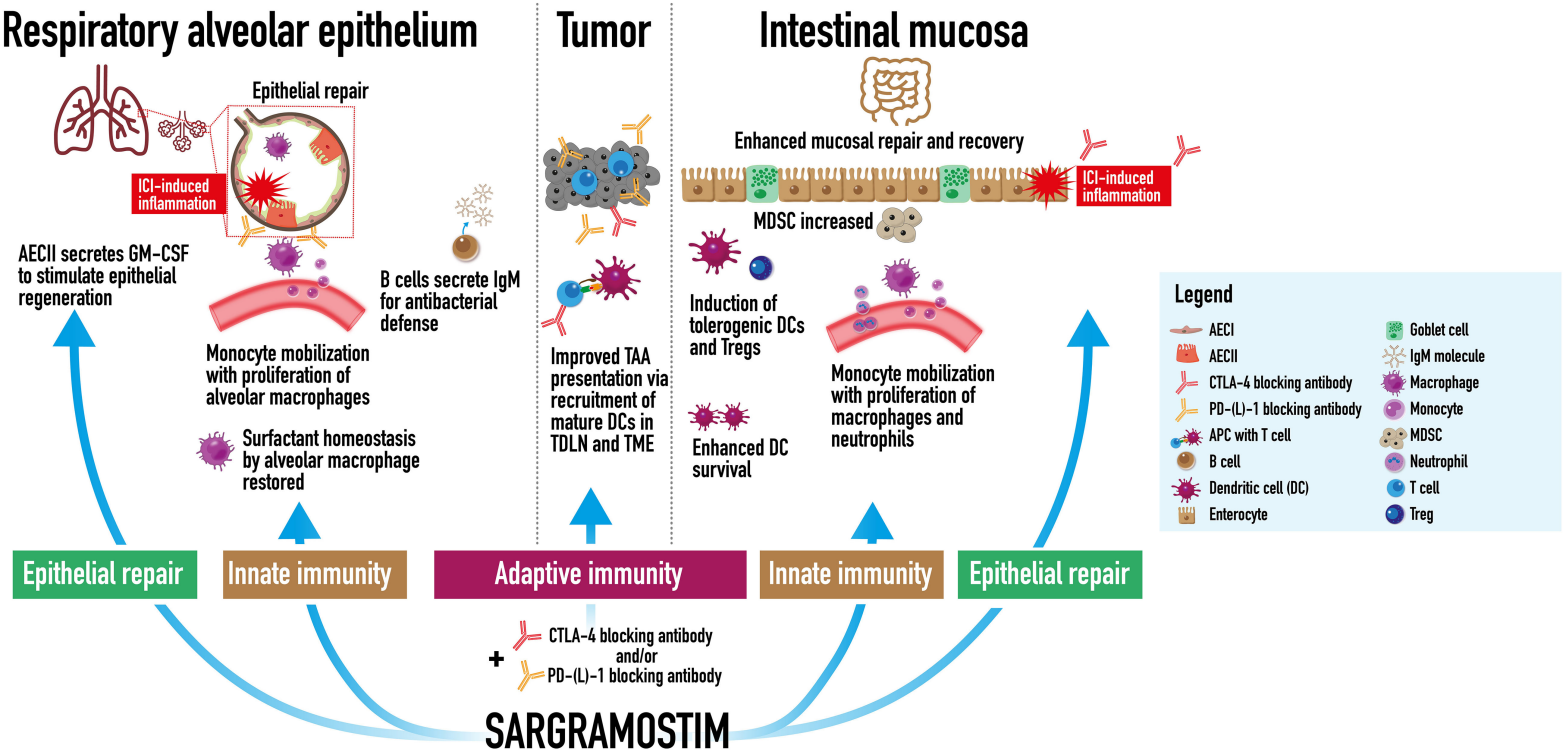
Possible antitumor and restorative activity of sargramostim in immune checkpoint inhibitor combination therapy. In checkpoint-induced pneumonitis, sargramostim (recombinant human granulocyte-macrophage colony-stimulating factor; rhu GM-CSF) may stimulate epithelial regeneration as modeled by endogenous GM-CSF-secreting Type II alveolar epithelial cell (AECII). Monocyte and alveolar macrophage mobilization and functional restoration of alveolar macrophages (which express programmed cell death protein 1 [PD-1] receptors) may contribute as well. Restoration of surfactant homeostasis by alveolar macrophages may support barrier renewal. Sargramostim may reinforce antibacterial defense via immunoglobulin M (IgM) secretion from B cells. Potential antitumor mechanisms of combination sargramostim and immune checkpoint inhibitor therapy may include improved tumor-associated antigen (TAA) presentation via recruitment of mature dendritic cells (DCs) in the tumor draining lymph node (TDLN) and tumor microenvironment (TME). In immune-mediated colitis, sargramostim mayenhance mucosal repair and recovery via induction of tolerogenic DCs and regulatory T cells (Tregs), and enhanced DC survival. Monocytes, macrophages, and neutrophils may be mobilized to bolster antibacterial defenses. Myeloid derived suppressor cells (MDSC) may be increased to enhance anti-inflammatory response. AECI, Type I alveolar epithelial cell; AECII, Type II alveolar epithelial cell; CTLA-4, cytotoxic T-lymphocyte associated protein-4; GM-CSF, granulocyte-macrophage colony-stimulating factor; ICI, immune checkpoint inhibitor; IgM, immunoglobulin M; PD-1, programmed death-1; PD-L1, programmed death ligand-1.

Current research in cancer immunotherapy includes the activities of endogenous cytokines, such as GM-CSF, and their potential to influence an antitumor immune response (21). While sargramostim has been used for 30 years for other purposes, recent research has focused on its immunomodulatory properties as discussed in the preceding sections (1). Intralesional rhu GM-CSF therapy increased tumor-infiltrating CD4^+^ T cell numbers and localized tumoricidal macrophages (233). CD4^+^ T cells play a major role in CD8^+^ T cell-mediated responses. An expanded macrophage response may lead to increased tumor-cell phagocytosis, antigen presentation and T-cell stimulation. Proposed mechanisms for increased survival in clinical studies with sargramostim include increased cytotoxic CD8^+^ T cell and dendritic cell recruitment to the tumor and sentinel lymph node, respectively (22–24). In addition, GM-CSF stimulation of increased metabolic capacity of mononuclear phagocytes may counteract the immunosuppressive potential of tumor associated myeloid cells (25). Interactions among these cells may lead to enhanced antitumor T cell priming and activation *via* increased dendritic cell tumor-associated antigen presentation (**Figure 3**).

ICI and sargramostim therapy link blockade of immune inhibitor mechanisms with increased immune cell activation which might boost antitumor responses in solid tumors (29). Correspondingly, in prostate cancer, sargramostim and ipilimumab combination therapy augmented tumor-reactive cytotoxic circulating CD8^+^ T cell responses (54). Furthermore, in melanoma, sargramostim and ipilimumab combination therapy increased overall survival (OS) and enhanced expression of inducible T-cell co-stimulator (ICOS) on CD4^+^ and CD8^+^ T cells over ipilimumab alone (28). Hence, addition of sargramostim appears to promote synergistic activation of key adaptive immune cells in antitumor immune responses.

### 6.2 Pathophysiology of immune-related adverse events

Until recently, the mechanisms of immune-mediated colitis were unclear. Biopsies from patients with colitis, in contrast to control patients, revealed a large population of proliferating, cytotoxic CD8^+^ T cells (234). Further analyses of inflamed tissue revealed that ICI colitis is characterized by expanded populations of IFN-γ- and granzyme B^+^-producing, cytotoxic CD8^+^ T cells, and to a lesser extent—expanded populations of Th1 skewed CD4^+^ T cells, and inflammatory macrophages (235–237). Based on clonal rearrangement analysis used to identify T cells and their progeny, these cytotoxic CD8^+^ T cells were determined to be derived from colon-resident memory cells, likely held in check by CLTA-4 and/or PD-1 receptors (238). These tissue-resident memory T cells are likely reactivated due to CLTA-4 and/or PD-1 blockade (237). It is hypothesized that reactivated tissue-resident memory T cells proliferate, become cytotoxic, and release IFN-γ. IFN-γ then may signal myeloid cells to amplify the inflammatory response and recruit T cells from the circulation, overwhelming Treg-mediated immunosuppression to damage colon tissue and impair barrier integrity.

In animal models, antitumor action of CTLA-4 blocking antibodies occurs *via* Treg depletion, cells that express high CTLA-4 levels (237). Human immune-mediated colitis biopsies indicate CTLA-4 Treg depletion is likely not a major mechanism of action of anti-CTLA-4 antibodies as the Treg population was preserved or expanded in post-CLTA-4 blockade biopsies.

Based on immune cells present in organs at homeostasis, the lungs share the same functional predictors of inflammatory toxicities as the GI tract (26). Both GI tract and lungs contain epithelial barriers colonized with microbiota, as well as a large population of tissue-resident memory T cells. Therefore, PD-1 pneumonitis may share mechanisms seen in colitis, including upregulation of inflammatory immune alveolar cells (TNF-α, IFN-γ, CD8+ cytotoxic T cells) and downregulation of important suppressor regulatory cells such as Tregs and alveolar macrophages that express PD-1 (27). Expression of CLTA-4 and PD-1 differs among different tissue cells and immune cells, which might explain why adverse event prevalence varies for immune checkpoint inhibitors between GI tract and lungs (229). Importantly, the addition of sargramostim to CTLA-4 blockade with ipilimumab reduced the incidence of GI and pulmonary adverse events, compared to ipilimumab alone in a phase 2 study of patients with metastatic melanoma (28).

#### 6.2.1 GM-CSF in lung and GI tract inflammatory disease

GM-CSF effects on immune cells have been documented in inflammatory diseases of the lung (*e.g.*, influenza virus and aPAP) and GI tract (*e.g.*, Crohn’s) and may play a role in checkpoint-induced pneumonitis and immune-mediated colitis (6, 11, 30). In the lung, type II alveolar epithelial cells secrete endogenous GM-CSF to stimulate alveolar epithelial regeneration (11, 118). As noted in **Emerging biology of GM-CSF**, GM-CSF induces activity, stimulates production, or functionally restores lung immune cells, *e.g.*, monocytes, alveolar macrophages, and B cells. As discussed in the aPAP section **(Autoimmune Pulmonary Alveolar Proteinosis (aPAP): a GM-CSF deficiency state**, alveolar macrophages play a major role in surfactant homeostasis, key to normal lung function (6). B cells secrete immunoglobulins, including IgM, as an antibacterial defense to protect the lung barrier (239). In the GI tract, GM-CSF has dual roles of increasing the number of MDSCs, and inducing monocytes, macrophages, neutrophils, and lamina propria dendritic cells (critical to Treg activation). Timing and extent of required or dysregulated immune response influence GM-CSF activity to either enhance anti-inflammatory activity or bolster antibacterial activity (30). Dendritic cell survival is also enhanced. Restoring or activating these pulmonary and GI cells after injury or infection (*e.g.*, potentially caused by dysregulated immunity), may contribute to epithelial cell regeneration and bacterial defense resulting in mucosal healing and fortification (**Figure 3**) (1).

### 6.3 Anti-tumor effects and irAE clinical investigations and gaps

Clinical combination studies of ICIs and sargramostim have shown benefit in metastatic melanoma and prostate cancer (only melanoma studies detailed here) (28, 29, 54–56, 240). The adverse event data are reported in **Supplement Table S1**. There were 3 author-reported grade 5 adverse events (death) in these clinical trials. All studies are of sargramostim in combination with ICI therapy.

In a phase 2 study, Hodi et al. (28) reported longer survival (17.5 months vs 12.7 months, *p*=0.01) and reduced grade 3-5 adverse events (45% vs 58%, p=0.04), for patients with unresectable stage III or IV melanoma treated with ipilimumab 10mg/kg plus sargramostim 250 μg subcutaneously days 1 to 14 of 21-day cycles (n=123) vs ipilimumab alone (n=122). Efficacy results were similar in two much smaller, non-randomized ipilimumab/sargramostim melanoma treatment studies wherein most patients had poor prognostic characteristics such as brain and liver metastases (55, 56). Patients with brain metastases are often excluded from clinical trials, however, they were included in the following studies which reported combination treatment benefit. A single institution, retrospective analysis (N=32) examined combination ipilimumab 3 mg/kg and sargramostim 250 μg/day for 14 days in 21-day cycles for 4 cycles (55). These authors reported overall disease control ≥ 12 weeks in 50% by response evaluation criteria in solid tumors (RECIST) and 44% by immune-related response criteria. Median OS was 41 weeks with an overall incidence of immune-related adverse events of 31.3% with 9.4% grade 3-4 events. A separate prospective, single-arm phase 2 trial (N=22) examined ipilimumab 10 mg/kg with sargramostim 125 μg/m^2^/day for 14 days in 21-day cycles, followed by sargramostim alone, then maintenance therapy of ipilimumab with sargramostim every 3 months for up to 2 years (56). Authors reported disease control in 41% of patients at 24 weeks. Median OS was 21.1 months, and grade 3-4 adverse events occurred in 41% of patients.

The ongoing ECOG-ACRIN EA6141 phase 2-3 trial is evaluating the combination of ipilimumab, nivolumab and sargramostim, compared with ipilimumab and nivolumab, in patients with unresectable stage III and stage IV melanoma. The trial advanced to the phase 3 portion after meeting prespecified efficacy and safety thresholds in the lead-in phase 2 portion (57). Trials continue to demonstrate the benefit of combination immunotherapy in melanoma and other diseases. However, this effect comes at a high toxicity cost for patients, making this exploration of triplet therapy incredibly important to the oncology field.

In addition to efficacy, sargramostim can act as an immune modulator to potentially attenuate or avoid immune-related adverse events (**Figure 3**). Randomized controlled trials primarily focusing on immune-related adverse event prevention and/or treatment are lacking. In patients with dysregulated immunity of the GI tract or lung (Crohn’s disease or aPAP), sargramostim has been shown to be more effective than placebo at inducing disease control (39, 241). In the phase 2 trial in melanoma discussed above, Hodi et al. (28) reported combination sargramostim and ipilimumab therapy led to fewer GI and pulmonary toxicities and improved survival compared to ipilimumab alone. Treatment paradigms have shifted since completion of this study and ipilimumab doses less than 10mg/kg are now used due to treatment-limiting toxicity of higher ipilimumab doses; thus, additional studies are warranted to verify these findings with the current treatment regimens.

### 6.4 Potential future developments in anti-tumor effects and irAE

Guidelines are evolving to include more combination ICI therapy options. Investigators for the phase 3 CheckMate 067 trial recently reported 6.5-year OS data for patients with stage III/IV melanoma (242). 49% of patients treated with nivolumab/ipilimumab (n=314) versus 42% with nivolumab alone (n=316), or 23% with ipilimumab alone (n=315) achieved a durable OS response. Duration of response for study patients at 6.5 years for nivolumab/ipilimumab dramatically exceeded that of single agent use of ipilimumab or nivolumab (61.9-NR months *vs* 8.8,-47.4 months *vs* 45.7-NR, respectively). However, patients assigned to the nivolumab/ipilimumab arm experienced higher rates of autoimmune toxicities than patients treated with the single agent immunotherapy agents. The ongoing study EA6141 will assess if the addition of sargramostim to the nivolumab/ipilimumab combination corroborates and improves results obtained from CheckMate 067 (57). The study will assess the triplet combination of sargramostim/nivolumab/ipilimumab also in the alleviation of toxicities which may make therapy more tolerable and greatly expand the patient population able to benefit from this therapy.

EA6141 and CheckMate 067 exclude melanoma patients with poor prognostic factors like active or untreated brain metastases (57, 242). CheckMate 204 focused on the effects of nivolumab/ipilimumab combination therapy on melanoma brain metastases (243). Follow-up CheckMate 204 report noted a 3-year OS of 71.9% for asymptomatic (n=101) and 36% for symptomatic (n=18) patients (244). An important clinical question to answer for patients with poor prognostic factors would be the impact of adding sargramostim to combination ICI therapy (*i.e.*, nivolumab/ipilimumab) on patient outcomes and rates of severe toxicity.

Investigations are underway to identify biomarkers with predictive utility for benefit from sargramostim. One strategy examines blood monocyte HLA-DR (human leukocyte antigen-DR isotype, a humanized major histocompatibility complex [MHC] II) expression on CD14^+^ immunosuppressive peripheral blood mononuclear cells, also known as MDSCs (25). This cell population is continuously renewed and reflects functional capacity of these cells, including their ability to phagocytose, to digest and present tumor antigens to T lymphocytes, as well as to support those lymphocytes with cytokines. Low HLA-DR expression has been proposed to be a surrogate marker of immunoparalysis in multiple clinical conditions, including sepsis where it is associated with worse patient outcomes (245). Equally, elevated monocytes with low HLA-DR expression have been correlated with worse outcomes for patients with melanoma treated with ipilimumab, among other cancers and therapies (246). In a number of different disorders as mentioned in **Wound healing and risk of GM-CSF deficiency** and **Immune responses to infection and risk of GM-CSF insufficiency**, sargramostim therapy increases HLA-DR expression and reverses immunoparalysis (65, 217) justifying further investigation in the context of cancer immunotherapy.

Additional trials are needed to evaluate immune-related adverse event risk factor prediction and prevention/reduction strategies, as well as the impact of immune-related adverse event management on ICI efficacy. Lozano et al. (247) reported, regardless of organ system, increased circulating activated CD4^+^ memory T cell numbers and increased T cell receptor diversity in melanoma correlate with an increased risk for severe immune-related adverse event development. Uncovering mechanisms responsible for organ-specific immune-related adverse events may enable expanded use of immunomodulatory agents in other disease states (*e.g.*, GM-CSF autoantibodies in aPAP or Crohn’s disease) (125, 248). Future study designs might incorporate serial tissue biopsies to map toxicity development, identify potential biomarkers, and define the immune cell signaling involved in checkpoint-induced pneumonitis and colitis. These research goals could be incorporated into randomized controlled trials that measure immune-related adverse events with immunomodulatory agents like sargramostim.

Supported by Hodi et al. (28), the anti-cancer potential of sargramostim may be additive to or synergistic with ICIs to boost efficacy in patients with metastatic melanoma. Results from ICI combination studies are encouraging and may help answer the unmet need of low cure rates in melanoma and other cancers. Combination sargramostim and the PD-1 inhibitor, pembrolizumab, is being studied in melanoma [NCT04703426 (249)], biliary cancer [NCT02703714 (250)], and NSCLC [NCT04856176 (251)]. These trials and other investigations in renal cell carcinoma, and head and neck cancers aim to better understand the role that sargramostim plays in the generation of antitumor immune responses as well as attenuating toxicity.

## 7 Discussion

GM-CSF deficiency disease classification and correlation with mononuclear phagocyte dysfunction has led to investigations of sargramostim in aPAP, infection, wound healing, anti-cancer treatment, and irAE amelioration. Within these disease treatment paradigms, further investigations of biomarkers and/or biopredictors such as HLA-DR status in combination with sargramostim may allow for efficient patient stratification and individualized treatment approaches in order to improve outcomes (172, 173, 217, 245). Ongoing investigations in other disease states and infections [*e.g.* nontuberculosis mycobacteria (252), pulmonary aspergillus (176), *C. gattii* cryptococcosis (186), and respiratory syncytial virus (112, 253)] further highlight the wide potential role of immunomodulatory agents such as sargramostim. Specifically, for neurodegenerative diseases, early investigations suggest innate immune modulation and illustrate GM-CSF/sargramostim potential to ameliorate symptoms and pathology of Alzheimer’s and Parkinson’s Diseases, among others (33–36). We await results of such on-going clinical trials.

## Author contributions

HL, conceptualization, methodology, data curation, supervision, writing- original draft and multiple revised drafts. KP and CR, methodology, data curation, supervision, writing- original draft and multiple revised drafts. TW, EL, EB, MD, DA, RP, and TB, writing-reviewing-editing. RG and ER, supervision and reviewing-editing. All authors contributed to the article and approved the submitted version.
