# Supplementary material for: Recombinant GM-CSF for diseases of GM-CSF insufficiency: Correcting dysfunctional mononuclear phagocyte disorders
Source: Front Immunol. 2023 Jan 5;13:1069444. doi: 10.3389/fimmu.2022.1069444 (PMC9850113; doi:10.3389/fimmu.2022.1069444)
Supplement: Supplementary file 1 [file DataSheet_1.docx]

Supplementary Material

Recombinant GM-CSF for Diseases of GM-CSF Insufficiency: Correcting Dysfunctional Mononuclear Phagocyte Disorders

Hillard M. Lazarus, MD^*^, Katherine Pitts, PharmD, Tisha Wang, MD, Elinor Lee, MD, PhD, Elizabeth Buchbinder, MD, Michael Dougan, MD, PhD, David G. Armstrong, DPM MD PhD^8^, Robert Paine III, MD, Carolyn E. Ragsdale, PharmD, Timothy Boyd, PhD, Edwin P Rock, MD, PhD, Robert Peter Gale, MD, PhD

*** Correspondence:** Corresponding Author: [hillard.lazarus@case.edu](mailto:hillard.lazarus@case.edu)

# Table S1: Adverse event data reported in rhu GM-CSF clinical studies

| **Author/Year*** | **Study Design** | **rhu GM-CSF Treatment** | **Reported Adverse Events** |
| --- | --- | --- | --- |
| **aPAP** | | | |
| Trapnell 2020 (1) | Prospective, randomized trial (N=138) | Molgramostim 300 μg inhaled daily, continuous or intermittently (every other week) x 24 weeks or placebo | - Double-blind treatment period   - AEs were similar in the three arms except for the percentage of patients with chest pain, which was higher in the continuous-molgramostim group (22%) compared to the intermittent-molgramostim group (4%), and placebo (2%)   - AEs reported in patients in the continuous molgramostim arm included cough (n=15; 32.6%), chest pain (n=10; 21.7%), nasopharyngitis (n=7; 15.2%), headache (n= 6; 13%), dyspnea (n=5; 10.9%), productive cough (n=4; 8.7%), pain in extremity (n=4; 8.7%), progression of aPAP (n=3; 6.5%), weight increase (n=3; 6.5%), nausea (n=3; 6.5%), pyrexia (n=2; 4.3%), back pain (n=2; 4.3%), arthralgia (n=2; 4.3%), chest discomfort (n=1; 2.2%), peripheral edema (n=1; 2.2%)     - Serious AEs included progression of aPAP (n=3; 6.5%), bacterial pneumonia, cough, dyspnea, laryngeal edema, respiratory failure, pneumonia, respiratory tract infection, aphasia, epilepsy (n=1 for each; 2.2%)   - AEs reported in patients in the intermittent molgramostim arm included cough (n=12; 26.7%), nasopharyngitis (n=10; 22.2%), headache (n=7; 15.6%), dyspnea (n=7; 15.6%), diarrhea (n=6; 13.3%), progression of aPAP (n=5; 11.1%), weight increase (n=5; 11.1%), productive cough (n=3; 6.7%), pyrexia (n=3; 6.7%), back pain (n=3; 6.7%), chest discomfort (n=3; 6.7%), peripheral edema (n=3; 6.7%), URTI (n=3; 6.7%), chest pain (n=2; 4.4%), nausea (n=1; 2.2%)     - Serious AEs included progression of aPAP (n=3; 6.7%), bacterial pneumonia (n=1; 2.2%), diverticulitis (n=1; 2.2%) - Open-label treatment-extension period:   - AEs reported in patients in the intermittent molgramostim arm included nasopharyngitis (n=24; 18.5%), cough (n=11; 8.5%), progression of aPAP (n=7; 5.4%), rash (n=6; 4.6%), bronchitis (n=5; 3.8%), URTI (n=5; 3.8%), pneumonia (n=5; 3.8%), dyspnea, peripheral edema, back pain, CRP increased (n=4 for each; 3.1%)     - Serious AEs included progression of aPAP (n=5; 3.8%), pneumonia (n=2; 1.5%), nasopharyngitis, bronchitis, influenza, bacterial pneumonia, urinary tract infection, oral candidiasis, lung infection, esophageal candidiasis, pulmonary tuberculosis, weight decrease, cataract, prostatitis, cholelithiasis, breast cancer, squamous cell carcinoma of the tongue (n=1 for each; 0.8%) |
| Tazawa 2019 (2) | Prospective, phase 2, randomized trial (N=64) | Sargramostim 125 μg inhaled twice daily x 7 days, every other week x 24 weeks or placebo | - AEs were similar in both arms - AEs reported in patients in the sargramostim arm included nasopharyngitis (n=12; 36.4%); URTI (n=8; 24.2%); pyrexia, headache, γ glutamyltransferase increased (n=3 for each; 9.1%); dental caries, influenza, pharyngitis, sinusitis, backpain, alveolar proteinosis (n=2 for each; 6.1%); malaise, bronchitis, hypertension, cough, insomnia, pruritis, AST increased, glucose urine, WBC decreased (n=1 for each; 3.0%) - Serious AEs were observed in 18.2% of patients in the sargramostim arm (ileus, congestive heart failure, pneumothorax, lacunar infarction, breast cancer, and worsening of aPAP plus influenza type A infection; n=1 of each) |
| Campo 2016 (3, 4) | Prospective, phase 2, randomized trial (N=18) | WLL followed by inhaled sargramostim 250 μg inhaled daily every other week x 12 weeks, then 250 μg daily x 2 consecutive days every 2 weeks x 6 months or WLL alone | - Authors reported inhaled sargramostim was well tolerated and safe; no additional details on AEs were provided |
| Tazawa 2010 (5) | Prospective, phase 2, crossover, self-controlled, open-label trial (N=50) | - Observation period x 12 weeks - Sargramostim High dose period: 125 μg   inhaled twice daily on days 1–8, no therapy on days 9–14 x six 2-week cycles (induction therapy)   - Sargramostim Low dose period: 125 μg inhaled daily on days 1–4, no therapy on days 5–14 x six 2-week cycles (maintenance therapy) | - AEs were reported in 17.9% of the patients in the sargramostim arm (fever, otitis media, gastric ulcer, URTI, diarrhea, pneumonia, and tuberculous lymphadenitis; n=1 for each) - Of these, sargramostim-related AEs included grade 2 pneumonia (n=1) and grade 2 tuberculous lymphadenitis (n=1) - No serious AEs were reported |
| **Immune Response to Infections** | | | |
| Paine 2022 (6) | Prospective, randomized, open-label trial (N=122) | Sargramostim 125 μg inhaled twice daily x 5 days plus SOC vs SOC | - Authors reported inhaled sargramostim was well tolerated - Treatment emergent AEs were similar in both arms (68% sargramostim arm vs 71% SOC arm) - Sargramostim-related treatment emergent AEs included dry mouth (n=1), hyponatremia (n=1), cough (n=1), chest discomfort and throat irritation (n=1), and hypoxia (n=1) - No serious AEs were related to sargramostim |
| Bosteels 2022 (7) | Prospective, randomized, open-label trial (N=81) | Sargramostim 125 μg inhaled twice daily x 5 days plus SOC vs SOC | - AEs occurred in 75% of patients in the sargramostim arm and 81% in the SOC arm. Serious AEs occurred in 15% of patients in the sargramostim arm and 10% in the SOC arm. Incidence of events was generally similar between arms, except more epistaxis occurred in the sargramostim arm (20% vs 5%). - AEs reported in the sargramostim arm included epistaxis (n=8; 20%), infectious disorder other than COVID-19 (n=7; 17.5%), constipation (n=3; 7.5%), acute kidney injury (n=2; 5%), cardiac disorder (n=2; 5%), thrombosis (n=1; 2.5%), and abnormal liver function (n=1; 2.5%) |
| Herold 2014 (8) | Single arm compassionate use study (N=6) | Sargramostim 125 µg inhaled every 48 hours vs historical controls (n=4) | - Authors reported no organ toxicities related to sargramostim treatment |
| Paine 2012 (9) | Prospective, phase 2 randomized, double-blind trial (N=130) | Sargramostim 250 µg/m^2^ IV infusion daily x 14 days vs placebo | - Serious AEs were similar in both arms - Serious AEs in the sargramostim arm included pulmonary AEs (n=11), sepsis or multiorgan system failure (n=11), and infection (n=7) |
| Pinder 2018 (10) | Prospective, phase 2, randomized, single-blinded trial (N=38) | Sargramostim 3 µg/kg SQ injection daily x 4 days vs placebo | - AEs reported in the sargramostim arm included fever, thrombocytopenia, raised platelet count, raised hepatic transaminases, internal jugular vein thrombus at central vein catheter insertion site, intestinal obstruction - 4 patients in the sargramostim group died, but deaths not considered by authors unexpected or potentially related to study drug |
| Rosenbloom 2005 (11) | Prospective, randomized, unblinded trial (N=40) | Sargramostim 125 µg/m^2^ continuous IV infusion over 72 hours (equivalent to 3 µg/kg/day) vs placebo | - Authors reported no detectable exacerbation of sepsis-related or *de novo* organ failure, no increase in incidence of progression to circulatory shock, or other deleterious AE |
| Presneill 2002 (12) | Prospective, phase 2, randomized, double-blind trial (N=18) | Molgramostim 3 µg/kg IV infusion daily x 5 days + SOC or placebo + SOC | - Authors reported molgramostim was generally well tolerated (9 of 10 patient courses administered to completion without toxicity greater than WHO Grade 1) - Serious AEs included transient reversible oliguria (n=1, WHO Grade 3 renal toxicity) |
| Hall 2011 (13) | Prospective, randomized, open-label trial (N=14) | Sargramostim 125 µg/m^2^ IV infusion daily x 7 days vs SOC | - Authors reported no AEs related to sargramostim treatment |
| Meisel 2009 (14) | Prospective, randomized, double-blind trial (N=38) | Sargramostim 4 µg/kg SQ injections daily x 5 days vs placebo  On day 6: sargramostim increased to 8 µg/kg/day (if HLA-DR ≤15,000 mAb/cell at day 5) or maintained at 4 µg/kg/day (if HLA-DR > 15,000 mAb/cell) | - Authors reported no AEs related to sargramostim treatment |
| Bilgin 2001 (15) | Prospective, randomized trial (N=60) | Molgramostim 5 µg/kg SQ injections daily x 7 days vs SOC | - Authors reported molgramostim as well tolerated with no adverse reactions; no additional details on AEs were provided |
| **Wound Healing** | | | |
| Da Costa 1997 (16) | Prospective, randomized trial (N=25) | rhu GM-CSF 400 μg one-time perilesional SQ injection or placebo | - Authors reported more frequent itching of the wound area for a longer number of days after injection in the molgramostim arm - No serious AEs or changes in the hematological and biochemical parameters studied were reported |
| Da Costa 1999 (17) | Prospective, randomized trial (N=61) | Molgramostim 200 μg or 400 μg perilesional SQ injections weekly x max 4 weeks or until wound closure + SOC or SOC | - AEs occurred in 38% of patients in the 200 μg molgramostim arm, 26% in the 400 μg molgramostim arm, and 9% in the placebo arm - Treatment-emergent AEs reported in the 200 μg molgramostim arm included lumbar pain (n=5; 24%), malaise (n=2; 10%), vagal reaction (n=2; 10%), local pain (n=1; 5%), lipothymia (n=1; 5%), chills (n=1; 5%) - AEs in the 400 μg molgramostim arm included malaise (n=3; 17%), local pain lumbar pain, vagal reaction, abdominal pain (all n=1; 6%) - Authors reported significant elevations in leukocyte and neutrophil counts, and prothrombin time in both 200 μg and 400 μg arms compared to placebo but judged differences to be clinically non-significant - No AEs were considered serious or life-threatening |
| Bianchi 2002 (18) | Prospective trial (N=5) | Molgramostim 5 μg/mL topical solution, 1-2mL applied topically three times daily x 1 week, then daily x 4 months | - Authors reported no clinical AEs or blood cell count abnormalities with molgramostim - Authors note a preference of topical administration *via* sprinkling samples of the GM-CSF working solution vs intradermal perilesional injections to avoid a combination of side effects observed in some patients (severe lumbar pain, tachypnea, hyperhidrosis that lasted 20-30 minutes and resolved without any treatment) |
| Karlafti 2018 (19) | Case report (N=1) | rhu GM-CSF 400 μg patch applied topically and injected once every 15 days x 2 months | - Authors reported no white blood cell count abnormalities with rhu GM-CSF treatment - No additional details on AEs were provided |
| Chi 2015 (20) | Prospective, randomized trial (N=30) | rhu GM-CSF 100 μg/10 g impregnated topical gel or placebo daily | - AEs in the rhu GM-CSF arm included fever/runny nose (n=3) and diarrhea (n=1) - Authors reported no significant difference between routine measurements of blood levels, urine parameters, liver function, and kidney function before rhu GM-CSF and 3 days after |
| **Anti-Cancer Potential and Mitigation of ICI irAE** | | | |
| Fong 2009 | Prospective, phase 1 trial (N=24) | Sargramostim 250μg/m^2^ SQ injections daily on days 1-14 of a 28-day cycle plus escalating doses of ipilimumab (0.5mg/kg, 1.5mg/kg, 3mg/kg) | - Grade 3 AEs reported with varying doses of ipilimumab and fixed-dose sargramostim include CVA, rash, pan-hypopituitarism, temporal arteritis, diarrhea (n=1 for each). - No Grade 5 AEs reported. |
| Kwek 2015 (21) | Prospective, phase 1b trial (N=42) | Sargramostim 250 μg/m^2^ SQ injections daily on days 1-14 of a 28-day cycle plus escalating doses of ipilimumab IV (0.5mg/kg, 1.5mg/kg, 3mg/kg, 5mg/kg, 10mg/kg) | - Grade 3-5 AEs reported with varying doses of ipilimumab and fixed dose sargramostim include CVA (n=2), fatigue (n=5), rash (n=4), diarrhea (n=4), atrial fibrillation (n=2), panhypopituitarism, **PE (Grade 5)**, elevated troponin, angina, temporal arteritis, DVT (n=1 for each) |
| Hodi 2014 (22) | Prospective, phase 2, randomized open-label trial (N=245) | Sargramostim 250 μg SQ injection on days 1-14 of a 21-day cycle plus ipilimumab IV 10mg/kg every 3 weeks x 4 doses, then every 12 weeks thereafter vs ipilimumab alone | - Grade 3-5 AEs occurred in 44.9% of patients in sargramostim arm and 58.3% of patients in ipilimumab alone arm (p = 0.04). - Notable AEs categories for differences were gastrointestinal (16.1% vs 26.7%; p = 0.05) and pulmonary (0 vs 7.5%; p = 0.003) toxicities in the sargramostim arm and ipilimumab alone arm, respectively. Colonic perforation occurred in 1.7% of patients in the sargramostim combination arm vs 5.8% in ipilimumab alone arm. - **Grade 5 AEs in the sargramostim arm included colonic perforation (n=1) and cardiac arrest (n=1)** |
| Chen 2018 (23) | Meta-analysis of 6 trials (N=445) | Comparison of combination therapy (ipilimumab plus sargramostim, varying doses) vs ipilimumab alone (varying doses) | - Fewer high-grade AEs occurred with combination therapy (ipilimumab plus sargramostim) than ipilimumab alone, especially the incidence of colonic perforation - Authors report a higher risk of AEs with combination ipilimumab 10 mg/kg and sargramostim 250 μg/m^2^ therapy for all-grade diarrhea and colitis compared to combination of ipilimumab 10 mg/kg and sargramostim 125 μg/m^2^ therapy. - Authors report a lower risk of AE with combination ipilimumab 10 mg/kg and sargramostim 250 μg/m^2^ therapy for all-grade nausea and fatigue than to the combination ipilimumab 10 mg/kg and sargramostim 125 μg/m^2^ therapy. - Subgroup analysis of high-grade AE showed combination ipilimumab 10 mg/kg and sargramostim 250 μg/m^2^ therapy could bring higher risk of nausea, colitis, fatigue than combination ipilimumab 10 mg/kg and sargramostim 125 μg/m^2^ therapy. |
| Kwek 2016 (24) | Prospective, phase 2 trial  (N=22) | - Sargramostim 125 μg/m^2^ SQ injection on days 1-14 of a 21-day cycle plus ipilimumab IV 10 mg/kg every 3 weeks x 4 cycles, then sargramostim 125 μg SQ injection on days 1-14 of a 21-day cycle alone x 4 cycles. - Maintenance therapy began at month 6 with combination therapy, every 3 months for up to 2 years or until disease progression or unacceptable toxicity | - Grade 3-4 AEs occurred in 41% of patients treated with ipilimumab and sargramostim. - Authors reported no treatment-related deaths |
| Luke 2015 (25) | Retrospective review (N=32) | Sargramostim 250 μg SQ injection on days 1-14 of a 21-day cycle plus ipilimumab IV 3 mg/kg every 3 weeks x 4 doses | - Grade 3-4 irAEs occurred in 9.4% of patients which included colitis (n=2; 6.25%) and dermatitis (n=1; 3.12%) - irAEs occurred in 31.3% of patients - Authors reported no treatment-related deaths |

^AEs: adverse events; aPAP: autoimmune alveolar pulmonary proteinosis; AST: aspartate aminotransferase; COVID-19: coronavirus disease of 2019; CRP: c-reactive protein; CVA: cerebrovascular accident; DVT: deep vein thrombosis; HLA: human leukocyte antigen; irAE: immune-related adverse event; IV: intravenous; mAB: monoclonal antibodies; PE: pulmonary embolism; rhu GM-CSF: recombinant human granulocyte-macrophage colony-stimulating factor; SOC: standard of care; SQ: subcutaneous; URTI: upper respiratory infection; WBC: white blood cell; WLL: whole lung lavage; WHO: World Health Organization^

^*The following studies did not report adverse event data: Brem 2018; Cianfarani 2006; Payne 2001; Robson 2000; Tazawa 2014; Wan 2015; Yan 2017^

**^Bolded text denotes treatment-related grade 5 adverse event (death). Note all patients were on combination therapies.^**

1. Trapnell BC, Inoue Y, Bonella F, Morgan C, Jouneau S, Bendstrup E, et al. Inhaled Molgramostim Therapy in Autoimmune Pulmonary Alveolar Proteinosis. *N Engl J Med* (2020) 383(17):1635-44. Epub 2020/09/09. doi: 10.1056/NEJMoa1913590.

2. Tazawa R, Ueda T, Abe M, Tatsumi K, Eda R, Kondoh S, et al. Inhaled GM-CSF for pulmonary alveolar proteinosis. *N Engl J Med* (2019) 381(10):923-32. Epub 2019/09/05. doi: 10.1056/NEJMoa1816216.

3. Campo I, Mariani F, Paracchini E, Kadija Z, Zorzetto M, Tinelli C, et al. Inhaled sargramostim and whole lung lavage (WLL) as therapy of autoimmune pulmonary alveolar proteinosis (aPAP). *Eur Respir J* (2016) 48(suppl 60):PA3870. doi: 10.1183/13993003.congress-2016.PA3870.

4. Campo I, Mariani F, Paracchini E, Kadija Z, Zorzetto M, Tinelli C, et al. Whole Lung Lavage Followed by Inhaled Sargramostim as Therapy of Autoimmune Pulmonary Alveolar Proteinosis. *Am J Respir Crit Care Med* 193(2016):A6438-A. doi: 10.1164/ajrccm-conference.2016.193.1_MeetingAbstracts.A6438.

5. Tazawa R, Trapnell BC, Inoue Y, Arai T, Takada T, Nasuhara Y, et al. Inhaled granulocyte/macrophage-colony stimulating factor as therapy for pulmonary alveolar proteinosis. *Am J Respir Crit Care Med* (2010) 181(12):1345-54. Epub 2010/02/20. doi: 10.1164/rccm.200906-0978OC.

6. Paine R, Chasse R, Halstead ES, Nfonoyim J, Park DJ, Byun T, et al. Inhaled Sargramostim (Recombinant Human Granulocyte-Macrophage Colony-Stimulating Factor) for COVID-19-Associated Acute Hypoxemia: Results of the Phase 2, Randomized, Open-Label Trial (iLeukPulm). *Military Medicine* (2022). doi: 10.1093/milmed/usac362.

7. Bosteels C, Van Damme KFA, De Leeuw E, Declercq J, Maes B, Bosteels V, et al. Loss of GM-CSF-dependent instruction of alveolar macrophages in COVID-19 provides a rationale for inhaled GM-CSF treatment. *Cell Reports Medicine*. doi: 10.1016/j.xcrm.2022.100833.

8. Herold S, Hoegner K, Vadász I, Gessler T, Wilhelm J, Mayer K, et al. Inhaled Granulocyte/Macrophage Colony–Stimulating Factor as Treatment of Pneumonia-associated Acute Respiratory Distress Syndrome. *American Journal of Respiratory and Critical Care Medicine* (2014) 189(5):609-11. doi: 10.1164/rccm.201311-2041le.

9. Paine R, 3rd, Standiford TJ, Dechert RE, Moss M, Martin GS, Rosenberg AL, et al. A randomized trial of recombinant human granulocyte-macrophage colony stimulating factor for patients with acute lung injury. *Crit Care Med* (2012) 40(1):90-7. Epub 2011/09/20. doi: 10.1097/CCM.0b013e31822d7bf0.

10. Pinder EM, Rostron AJ, Hellyer TP, Ruchaud-Sparagano MH, Scott J, Macfarlane JG, et al. Randomised controlled trial of GM-CSF in critically ill patients with impaired neutrophil phagocytosis. *Thorax* (2018) 73(10):918-25. Epub 20180731. doi: 10.1136/thoraxjnl-2017-211323.

11. Rosenbloom AJ, Linden PK, Dorrance A, Penkosky N, Cohen-Melamed MH, Pinsky MR. Effect of granulocyte-monocyte colony-stimulating factor therapy on leukocyte function and clearance of serious infection in nonneutropenic patients. *Chest* (2005) 127(6):2139-50. Epub 2005/06/11. doi: 10.1378/chest.127.6.2139.

12. Presneill JJ, Harris T, Stewart AG, Cade JF, Wilson JW. A randomized phase II trial of granulocyte-macrophage colony-stimulating factor therapy in severe sepsis with respiratory dysfunction. *Am J Respir Crit Care Med* (2002) 166(2):138-43. Epub 2002/07/18. doi: 10.1164/rccm.2009005.

13. Hall MW, Knatz NL, Vetterly C, Tomarello S, Wewers MD, Volk HD, et al. Immunoparalysis and nosocomial infection in children with multiple organ dysfunction syndrome. *Intensive Care Med* (2011) 37(3):525-32. Epub 2010/12/15. doi: 10.1007/s00134-010-2088-x.

14. Meisel C, Schefold JC, Pschowski R, Baumann T, Hetzger K, Gregor J, et al. Granulocyte–macrophage colony-stimulating factor to reverse sepsis-associated immunosuppression. *American Journal of Respiratory and Critical Care Medicine* (2009) 180(7):640-8. doi: 10.1164/rccm.200903-0363oc.

15. Bilgin K, Yaramiş A, Haspolat K, Taş MA, Günbey S, Derman O. A randomized trial of granulocyte-macrophage colony-stimulating factor in neonates with sepsis and neutropenia. *Pediatrics* (2001) 107(1):36-41. Epub 2001/01/03. doi: 10.1542/peds.107.1.36.

16. Da Costa RM, Jesus FM, Aniceto C, Mendes M. Double-blind randomized placebo-controlled trial of the use of granulocyte-macrophage colony-stimulating factor in chronic leg ulcers. *Am J Surg* (1997) 173(3):165-8. doi: 10.1016/s0002-9610(97)89589-x.

17. Da Costa RM, Ribeiro Jesus FM, Aniceto C, Mendes M. Randomized, double-blind, placebo-controlled, dose- ranging study of granulocyte-macrophage colony stimulating factor in patients with chronic venous leg ulcers. *Wound Repair Regen* (1999) 7(1):17-25. doi: 10.1046/j.1524-475x.1999.00017.x.

18. Bianchi L, Ginebri A, Hagman JH, Francesconi F, Carboni I, Chimenti S. Local treatment of chronic cutaneous leg ulcers with recombinant human granulocyte-macrophage colony-stimulating factor. *J Eur Acad Dermatol Venereol* (2002) 16(6):595-8. doi: 10.1046/j.1468-3083.2002.00526.x.

19. Karlafti E, Savopoulos C, Hatzitolios A, Didangelos T. Local use of granulocyte-macrophages colony stimulating factor in treatment of chronic diabetic neuropathic ulcer (Case Review). *Georgian Med News* (2018) (277):21-7.

20. Chi YF, Chai JK, Luo HM, Zhang QX, Feng R. Safety of recombinant human granulocyte-macrophage colony-stimulating factor in healing pediatric severe burns. *Genet Mol Res* (2015) 14(1):2735-41. Epub 20150331. doi: 10.4238/2015.March.31.3.

21. Kwek SS, Lewis J, Zhang L, Weinberg V, Greaney SK, Harzstark AL, et al. Preexisting Levels of CD4 T Cells Expressing PD-1 Are Related to Overall Survival in Prostate Cancer Patients Treated with Ipilimumab. *Cancer Immunology Research* (2015) 3(9):1008-16. doi: 10.1158/2326-6066.cir-14-0227.

22. Hodi FS, Lee S, McDermott DF, Rao UN, Butterfield LH, Tarhini AA, et al. Ipilimumab plus sargramostim vs ipilimumab alone for treatment of metastatic melanoma: a randomized clinical trial. *JAMA* (2014) 312(17):1744-53. doi: 10.1001/jama.2014.13943.

23. Chen P, Chen F, Zhou B. Comparisons of therapeutic efficacy and safety of ipilimumab plus GM-CSF versus ipilimumab alone in patients with cancer: a meta-analysis of outcomes. *Drug Design, Development and Therapy* (2018) Volume 12:2025-38. doi: 10.2147/dddt.s154258.

24. Kwek SS, Kahn J, Greaney SK, Lewis J, Cha E, Zhang L, et al. GM-CSF and ipilimumab therapy in metastatic melanoma: Clinical outcomes and immunologic responses. *Oncoimmunology* (2016) 5(4):e1101204. Epub 20151029. doi: 10.1080/2162402X.2015.1101204.

25. Luke JJ, Donahue H, Nishino M, Giobbie-Hurder A, Davis M, Bailey N, et al. Single Institution Experience of Ipilimumab 3 mg/kg with Sargramostim (GM-CSF) in Metastatic Melanoma. *Cancer Immunol Res* (2015) 3(9):986-91. Epub 20150505. doi: 10.1158/2326-6066.CIR-15-0066.
